# Cadmium Phytotoxicity, Tolerance, and Advanced Remediation Approaches in Agricultural Soils; A Comprehensive Review

**DOI:** 10.3389/fpls.2022.773815

**Published:** 2022-03-09

**Authors:** Usman Zulfiqar, Wenting Jiang, Wang Xiukang, Saddam Hussain, Muhammad Ahmad, Muhammad Faisal Maqsood, Nauman Ali, Muhammad Ishfaq, Muhammad Kaleem, Fasih Ullah Haider, Naila Farooq, Muhammad Naveed, Jiri Kucerik, Martin Brtnicky, Adnan Mustafa

**Affiliations:** ^1^Department of Agronomy, University of Agriculture Faisalabad, Faisalabad, Pakistan; ^2^College of Life Sciences, Yan’an University, Yan’an, China; ^3^Department of Botany, University of Agriculture Faisalabad, Faisalabad, Pakistan; ^4^Agronomic Research Institute, Ayub Agricultural Research Institute, Faisalabad, Pakistan; ^5^College of Resources and Environmental Sciences, Gansu Agricultural University, Lanzhou, China; ^6^Department of Soil and Environmental Science, College of Agriculture, University of Sargodha, Sargodha, Pakistan; ^7^Institute of Soil and Environmental Science, University of Agriculture Faisalabad, Faisalabad, Pakistan; ^8^Institute of Chemistry and Technology of Environmental Protection, Faculty of Chemistry, Brno University of Technology, Brno, Czechia; ^9^Department of Agrochemistry, Soil Science, Microbiology and Plant Nutrition, Faculty of AgriSciences, Mendel University in Brno, Brno, Czechia; ^10^Institute for Environmental Studies, Faculty of Science, Charles University in Prague, Prague, Czechia

**Keywords:** cadmium, contamination, abiotic stress, plant physiology and growth, remediation

## Abstract

Cadmium (Cd) is a major environmental contaminant due to its widespread industrial use. Cd contamination of soil and water is rather classical but has emerged as a recent problem. Cd toxicity causes a range of damages to plants ranging from germination to yield suppression. Plant physiological functions, i.e., water interactions, essential mineral uptake, and photosynthesis, are also harmed by Cd. Plants have also shown metabolic changes because of Cd exposure either as direct impact on enzymes or other metabolites, or because of its propensity to produce reactive oxygen species, which can induce oxidative stress. In recent years, there has been increased interest in the potential of plants with ability to accumulate or stabilize Cd compounds for bioremediation of Cd pollution. Here, we critically review the chemistry of Cd and its dynamics in soil and the rhizosphere, toxic effects on plant growth, and yield formation. To conserve the environment and resources, chemical/biological remediation processes for Cd and their efficacy have been summarized in this review. Modulation of plant growth regulators such as cytokinins, ethylene, gibberellins, auxins, abscisic acid, polyamines, jasmonic acid, brassinosteroids, and nitric oxide has been highlighted. Development of plant genotypes with restricted Cd uptake and reduced accumulation in edible portions by conventional and marker-assisted breeding are also presented. In this regard, use of molecular techniques including identification of QTLs, CRISPR/Cas9, and functional genomics to enhance the adverse impacts of Cd in plants may be quite helpful. The review’s results should aid in the development of novel and suitable solutions for limiting Cd bioavailability and toxicity, as well as the long-term management of Cd-polluted soils, therefore reducing environmental and human health hazards.

## Introduction

**FIGURE 1:**
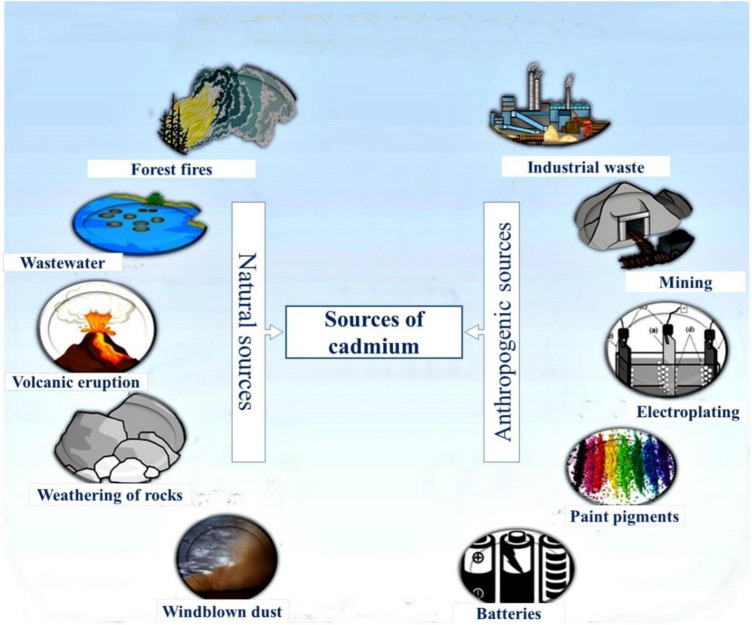
Sources of cadmium (Cd) contamination in the environment.

The presence of organic and inorganic pollutants in the environment leads to its deterioration, which has become a grave issue and is threatening the global ecosystem (Zulfiqar et al., 2019; Zeeshan et al., 2021). Enrichment of soil with toxic heavy metals such as cadmium (Cd), lead (Pb), arsenic (As), nickel (Ni), mercury (Hg), and chromium (Cr) causes serious hazards to plant life and human health. These potentially toxic elements are present at low concentrations in the environment (Palansooriya et al., 2020). High levels of these toxic metals are harmful to humans, plants, and animals (but not exceptionally) because of their persistent nature in the environment (Afzal et al., 2019). Cd is one of the most toxic heavy metals to living organisms (Chellaiah, 2018; Zulfiqar et al., 2021). Cd is an element ranked 7th in the list of 20 most toxic metals and classified as group 1 carcinogen (Jaishankar et al., 2014). It is one of the most perilous metals owing to its high toxicity and serious extent of bioaccumulation (Singh et al., 2020; Qianqian et al., 2022). Cd toxicity adversely affects the human body, and it accumulates in the kidneys and causes emphysema, renal tubular damage, and kidney stones (Mahajan and Kaushal, 2018). In minerals, it replaces calcium owing to similar charge, ionic radius, and chemical behavior (Kubier et al., 2019). Therefore, it can easily be transferred and stored in the human body (Hajeb et al., 2014). Cd toxicity causes severe liver damage and reduces the supply of calcium in the body (Lata et al., 2019). Moreover, Cd directly influences the regulation of Zn and Fe *via* ZIP, and NRAMP (Tanveer and Shabala, 2022). It is released to the environment through natural as well as anthropogenic systems. Among natural systems, weathering of Cd-containing rocks, forest fires, volcanic eruptions, and wastewater are the principal means (Liu et al., 2013; Manzoor et al., 2019). Anthropogenic activities are a source of Cd contamination, mainly including metallurgical works, mining, electroplating, paints, combustion emissions, and excessive use of fertilizers and pesticides (Singh et al., 2020; Haider et al., 2021a; Figure 1). It is readily soluble, and it is mobile compared to other metals; therefore, it is quickly taken by plants (Song et al., 2015). After uptake, Cd is translocated and accumulated in edible parts of plants (Adil et al., 2020).

Cadmium toxicity adversely affects plants by inhibiting carbon fixation and reducing chlorophyll synthesis and photosynthetic activity (Liu et al., 2018). Exposure to Cd causes physiochemical, morphological, and structural changes in plants such as chlorosis and inhibition of lateral root formation and stomatal density (Bari et al., 2019; Huybrechts et al., 2020). It induces osmotic stress in plants by reducing leaf relative water content, stomatal conductance, and transpiration (Rizwan et al., 2016). It also has harmful effects on uptake and transportation of mineral elements, resulting in yield decline (Chen et al., 2018a,b,c). Cd toxicity causes overproduction of reactive oxygen species (ROS) and results in damage to plant membranes and destruction of cell organelles (Abbas et al., 2017). Cd is a very toxic heavy metal that adversely affects a variety of physiological functions leading to stunted growth with ultimate yield penalty on field crops.

The bioavailability and toxicity of Cd depend on physical and chemical properties of soil (Violante et al., 2010). With decrease in soil pH, Cd is transformed from a fixed form to a readily mobile form that enhances its availability for plant uptake (Mondal et al., 2020). Therefore, understanding the physical and chemical properties of soil and the dynamics of Cd in soil is essential for reducing the toxicity caused by Cd. This review presents an overview of the adverse effects of Cd toxicity on plants, ultrastructural and oxidative damage, carbon metabolism, and yield formation. The dynamics of Cd in the rhizosphere, and soil factors affecting soil uptake are also discussed. Moreover, potential remediation strategies such as physical, chemical, and biological methods to decontaminate Cd from polluted soils are also highlighted. Furthermore, the use of different forms of organic materials and molecular techniques to reduce Cd uptake and accumulation are described.

## Cadmium Dynamics in Soil and the Rhizosphere

Biologically, Cd is not important for plants; however, it is easily acquired by plants because of micronutrients from the rhizosphere of soils (the soil-root interface) (Shahid et al., 2016). The presence of Cd has been observed in soils ranging between 0.07 to 1.1 mg kg^–1^ soil (World Health Organization [WHO], 2007). However, threshold level is approximately 100 mg kg^–1^ in agricultural soils (Asgher et al., 2015).

Cadmium (Cd) is primarily present as Cd ions or forming complexes, i.e., organic and inorganic in the soil solution. Both anionic and cationic forms of Cd exist in soils (Kabata-Pendias and Sadurski, 2004). Anionic forms are CdCl_3_^–^, Cd(OH)_3_^–^, Cd(OH)_4_^2–^, and Cd(HS)_4_^2–^, while cationic forms are CdCl^+^, CdOH^+^, CdHS^+^, and CdHCO^3+^. It has been found that 99% Cd is present in the soil solution as a free ionic form (Kabata-Pendias, 1993).

Several chemical reactions, namely, dissolution/precipitation, desorption/adsorption, and Cd ligand formation, affect the partitioning of Cd in soils. These processes are mainly influenced by ligands (organic and inorganic) (Shahid et al., 2014), redox conditions (Zhang et al., 2012), soil pH (Saeki and Kunito, 2012), metal contents, and temperature (Silber et al., 2012). Partitioning of Cd is vital in soil systems for regulation of Cd toxicity (Rizwan et al., 2017). Cd biogeochemical behavior depends on the concentration of free Cd ions in a soil medium (Shahid et al., 2016). Accumulation of Cd in plant root varies with Cd contents in the rhizosphere and plant type. Maize (*Zea mays* L.) showed more Cd accumulation in cell wall fraction than broad bean (*Vicia faba* L.) seedlings (Lozano-Rodriguez et al., 1997).

## Factors Affecting Cadmium Dynamics

Several factors like soil pH, cation exchange capacity (CEC), organic matter, microbial activities in the soil, and root exudates influence the bioavailability of Cd (Jung, 2008; Shahid et al., 2016). One of the crucial factors in the regulation of Cd partitioning and its bioavailability is soil pH (Yu et al., 2016). Cd exists in various chemical forms at varying soil pH levels. It has been observed that Cd solubility in the soil solution is primarily affected by acidic soil conditions. A change in Cd from immobile forms like carbonates and Mn and Fe oxides to better exchangeable forms allow free Cd phytoavailability and mobility (Qi et al., 2018). For the solubility of Cd in soil, pH 6 acts as a threshold point because of complex formation with organic matter and its adsorption on mineral surfaces (Sullivan et al., 2013). On the other hand, rise in pH increases its alkalinity, affecting Cd adsorption into soil particles. Yu et al. (2016) described that soil pH played a key role in acclimatization of Cd in rice grains. Enhanced soil pH imparts a negative influence on phytoavailability, as adsorption and precipitation of Cd decrease free Cd availability in the soil solution (Meng et al., 2018).

The bioavailability of Cd is influenced by soil organic matter (SOM) because of formation of various complexes with Cd in the soil solution. The bioavailability of Cd depends on SOM source, concentration, and chemical forms. In addition, SOM has a direct influence on Cd binding and its acclimatization. Kirkham (2006) reported that higher SOM causes more sorption potential, which is 30 times more than mineral soil. Biochar application greater than 10% reduces the bioavailability of Cd in plants through its immobilization in soil (Xiao et al., 2019). In another study, the application of biochar decreased Cd stress in wheat (*Triticum aestivum* L.) by reducing its bioavailability (Abbas et al., 2018). On the contrary, Yousaf et al. (2016) depicted that SOM content and uptake of Cd increased in wheat predominantly because of application of poultry manure, sewage sludge, and farmyard manure.

Cation exchange capacity of the soil strongly influences the mobility and bioavailability of Cd. In a study, binding of Cd to exchangeable and acid-soluble fractions occurred in loamy and loamy sand soils having small Cd contents, and was found to be related to SOM. However, Cd was bound to a reducible fraction followed by an exchangeable acid-soluble fraction in silt-clay soil (Gusiatin and Klimiuk, 2012). Hong et al. (2002) reported less Cd mobility due to its strong affinity with clay mineral surface, Fe–Al oxides, and humus in clayey soils. The bioavailability Cd is directly influenced by the occurrence of mineral ions in the soil solution. This is directly related to ionic strength, competition, and complexation for root or soil exchange sites. Additionally, there is an inverse relationship between ionic strength and bioavailability, as Cd extraction by plants is enhanced because of less ionic strength in growth media (Gothberg et al., 2004).

Soil microbial activity is found to enhance the availability of Cd through organic acid secretion and succeeding solubilization of Cd-bearing minerals (Ahmad et al., 2015). Soil amendments having Cd-solubilizing microbes like plant growth-promoting rhizobacteria (PGPRs) play an essential role in enhancing the bioavailability of Cd (Wu et al., 2020). In a study, Sangthong et al. (2016) depicted the ability of *Micrococcus* sp. TISTR2221 to modulate more uptake of Cd in the root and stem parts of maize plant under Cd stress. On the other hand, to reduce the toxicity of Cd in plants, microbes like PGPRs and arbuscular mycorrhizal fungi play a crucial role in restricting the uptake of Cd in roots. Reduction in Cd phytoavailability was found by soil bioaugmentation, causing immobilized and free Cd-resistant bacteria and fungi in the rhizosphere (Sharma and Archana, 2016).

**FIGURE 2:**
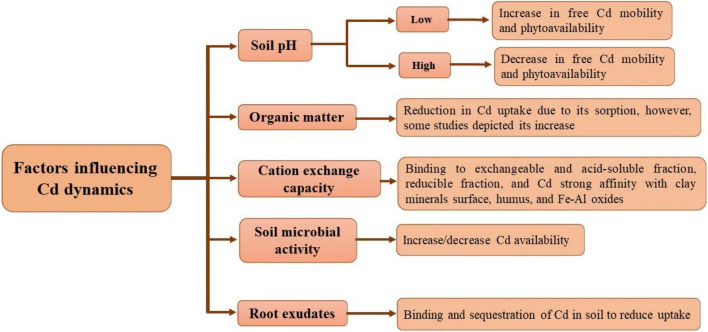
Factors affecting Cd dynamics in soils (conceived from Shahid et al., 2016).

Root exudates also impart a role in sequestration and binding of Cd in soils and protect plant roots from Cd toxicity in soils (Liao and Xie, 2004). Furthermore, Cd uptake is minimized by root exudates in plants (Sarwar et al., 2010). Factors affecting Cd dynamics are presented in Figure 2.

## Toxic Impacts of Cadmium on Plants

### Impact of Cadmium Toxicity on Plant Growth and Yield Formation

Cadmium accumulation in agricultural soils has become a primary concern of scientific factions because of Cd’s increasing concentration, which substantially reduces plant growth and yield (Romero-Puertas et al., 2004; Goix et al., 2014; Zhang et al., 2014). Cd toxicity in soils causes several harmful impacts on plants, i.e., overproduction of oxidative markers like ROS, free radicals, and lipid peroxidation which induces oxidative stress and ultimately causes reduction in the yield of crops (Qayyum et al., 2017; Hussain et al., 2018). Therefore, it is a necessity for the current era to overcome Cd toxicity for better health of humans and plants. To overcome the toxic impacts of Cd, following strategies could be useful such as introduction of plant species that can phytoremediate heavy metals by sequestration of the metals in their vegetative parts and avoidance of the introduction of these heavy metals to plants by control of anthropogenic activities and implementation of lawful strategies of countries and governments.

### Ultrastructural Changes Due to Cadmium Toxicity

Plants exhibit a different response when exposed to varying concentrations of Cd. Anatomical abnormalities mainly depend on plant species, exposure duration, uptake amount, sequestration, and localization in different parts (Shah et al., 2019). Cadmium is phloem-mobile and localized in any part of plants; it leads to reduction in biomass and yield; it causes chlorosis and even leaf fall that contradicts normal plant movements (Gallego et al., 2012).

Cadmium exposure causes considerable anatomical alterations in roots, stems, and leaves of *Ceratopteris pteridoides*. These alterations include closure of abaxial stomata, stomatal size reduction in leaves, scarification in tracheid walls, narrow xylem vessels, and disorganization in vascular bundles in roots and stems (Bora and Sarma, 2021). Trichome length, abaxial and adaxial density of stomata, and proportion of cortex were decreased in *Trigonella foenum* under Cd stress (Ahmad et al., 2005). Heterogeneity in vascular tissues of stems and leaves occurs in *Arundo donax* L. when exposed to Cd stress (Guo and Miao, 2010). Plants exposed to Cd cause severe impacts by reducing the size of parenchyma in leaves, disrupting the ultrastructure of chloroplasts, disorganizing vascular s organization, reducing epidermal tissue thickness, and exhibiting narrow xylem and phloem vessels. Heavy metal-tolerant plants can induce a plethora of mechanisms to reduce the noxious impacts of heavy metals by modification of microstructures. These modifications include an efficient vascular system by increasing a vascular bundle area for better translocation of water and food. These plants also develop a thick epidermis to conserve plenty of water in their bodies, which is covered with a waxy cuticle layer, and sequester a large amount of water in roots and shoots to prevent from translocation into the leaves. This prevention in uptake could modify the photosynthetic apparatus of plants. Some heavy metal-tolerant species also exhibit various avoidance mechanisms to prevent the entrance of heavy metals into roots. Effects of Cd on the ultrastructure of roots, stems, and leaves of different plant species are illustrated in Table 1.

**TABLE 1:** Ultrastructural/anatomical damages in different plant species due to cadmium (Cd) toxicity.

Plant species	Cd levels	Ultrastructural changes/damages	References
**At root level**			
*Gossypium hirsurum* L.	20.26 μM	Cd deposit between intercellular spaces of secondary phloem, and root periderm indicated adsorption and localization of Cd.	Chen et al., 2015
*Solanum tuberosum* L.	25 μM	Accumulation of Cd in root cells was higher than in the stem.	Xu et al., 2013
*Hordeum vulgare* L.	30 μM	Hyper accumulation of the Cd in root tissues as compared to its counterparts. Cd cause reduction of passage cells in the endodermis, thickened pericycle cell walls was assessed.	Alle et al., 2016
*Pteris vittata* L.	100 μM	Fewer numbers of root hairs, reduce apical meristem, reddish colored precipitates formed in root vacuoles.	Balestri et al., 2014
*Oryza sativa* L.	100 μM	Disintegration occurs in root cell walls and vascular tissues, brown granular deposits in the root exodermal cells, and prominence of root nucleoli.	Shah et al., 2013
*Miscanthus floridulus* L.	10 μM	Hyper accumulation of Cd in root cells.	Guo et al., 2016
*Zea mays* L.	0.1 mM	Extensive root area, large parenchyma, and cortical cells of roots.	Maksimović et al., 2007
*Aegiceras corniculatum* L.	4 g L^–1^	Root tissues decreased in the following order: endodermis > pith > xylem > epidermis and exodermis > phloem > cortex.	Li J. et al. (2019)

**At stem level**			
*Ceratopteris pteridoides* L.	60 μM L^–1^	Tracheids consist of pits in later walls, narrowing occur in the xylem and phloem vessels, vascular bundles disrupted in the form of aggregation, grana were dissolved and chloroplast form ellipsoidal shape.	Bora and Sarma, 2021
*Trigonella foenum* L.	50 μg g^–1^	Proportion of cortex and vasculature decreased, prominent alteration occur in the xylem and phloem.	Ahmad et al., 2005
*Arundo donax* L.	101 mg kg^–1^	Lower proportion of xylem, thin epidermal tissues, sclerification occurs in epidermis.	Guo and Miao, 2010

**At leaf level**			
*Pistia stratiotes* L.	12.8 mg L^–1^	Reduce proportion of aerenchyma in leaves.	Silva et al., 2013
*Avicennia schaueriana* L.	64 mg L^–1^	Disruption occurs in nuclear membranes, dense material deposit in the vascular bundles of parenchyma cells.	Mizushima et al., 2019
*Eucalyptus urophylla* L.	450 μM	Decline occurs in adaxial and abaxial epidermal thickness, palisade, and spongy parenchyma thickness.	da Silva Cunha et al., 2020
*Cicer arietinum* L.	0.1 mM	Reduce leaf thickness, abaxial and adaxial stomata closed	Liza et al., 2020
*Ceratopteris pteridoides* L.	60 μM L^–1^	Cause stomatal closure, narrow xylem vessels, disorganized chloroplast, and chloroplast components, excessive plastoglobules and large starch grains.	Bora and Sarma, 2021
*Populus deltoides* L.	8.14 mg kg^–1^	Size of palisade tissues decreased, adaxial epidermal cell size decreased.	Nikolić et al., 2017
*Salvia sclarea* L.	100 μM	Decline in epidermal cell size, osmiophilic granules embedded in cell vacuoles, loss of intercellular spaces, dense mesophyll cells seemed.	Dobrikova et al., 2021

### Oxidative Damages Due to Cadmium Toxicity

**FIGURE 3:**
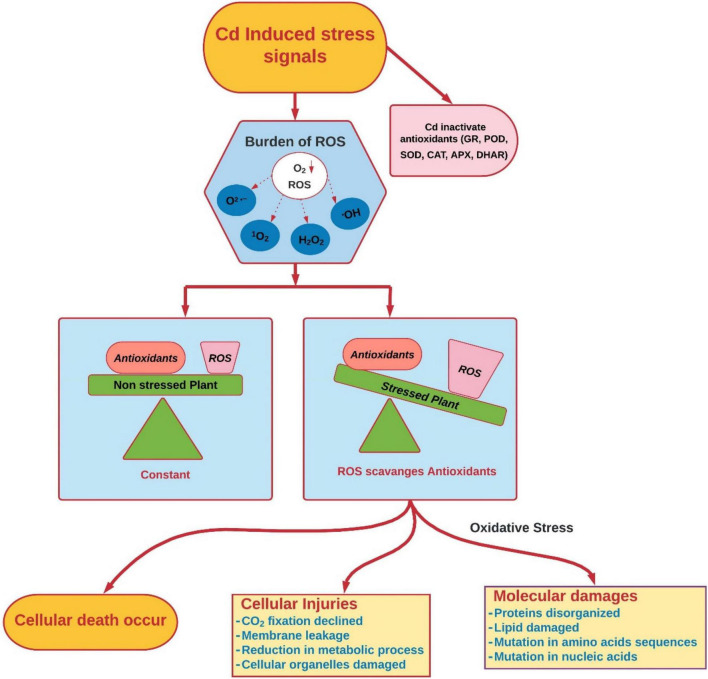
Cd-induced oxidative stress and damages in plants (conceived from Genchi et al., 2020; Shiyu et al., 2020). 1O_2_, singlet oxygen; ROS, reactive oxygen species; O_2_^•–^, superoxide anion; H_2_O_2_^•^, hydrogen peroxide; ^•^OH, hydroxyl radical; APX, ascorbate peroxidase; GR, glutathione reductase; POD, peroxidase; CAT, catalase; SOD, superoxide dismutase; DHAR, dehydro-ascorbate reductase.

Cadmium, like other HMs, induces oxidative damages by producing excessive H_2_O_2_ and lipid peroxidation in plants (Rizwan et al., 2019a; Shiyu et al., 2020; Yang et al., 2020; Figure 3). It is well-documented that Cd regimes induce the production of ROS (H_2_O_2_, O^–2^); these scavenge antioxidant enzymes (Kapoor et al., 2019; Hasanuzzaman et al., 2020; Unsal et al., 2020). Several studies suggest that Cd does not directly participate in ROS production but induces temporal oxidative damage to plants (Cuypers et al., 2010, 2016). Cellular ROS mainly comprise both free radicals and non-radicals (Hasanuzzaman et al., 2020). Free radicals include O^2^ •−, •OH, RO•, peroxyl radical (ROO•) and non-radicals, H_2_O_2_, 1O_2_, and ozone (O_3_) (Farooq et al., 2019; Maurya, 2020), while other non-radicals that exist in plants are excited carbonyls, hypochlorous acid (HOCl) and hydroperoxides (ROOH) (Kapoor et al., 2015). Because of oxidative damage, accumulation of thiobarbituric acid reactive substances (TBARSs) and malondialdehyde (MDA) occurs in, and results in electrolyte leakage under Cd stress (Younis et al., 2016).

Accumulation of Cd in plants occurs because of some significant mechanisms like (i) structural similarity with nutrients taken by roots (phosphorous and zinc), (ii) direct Cd influence on the sulfhydryl (–SH) group, which impairs protein structure, (iii) dislocation of essential cations from binding sites, (iv) disturbance of normal level of ROS and antioxidants, which consequently damage nucleic acids, lipids, proteins, cellular pigments, and essential nutrients (Hossain et al., 2012; Choppala et al., 2014; Singh et al., 2016). Cd induces oxidative stress in several plant species, i.e., *Phyllostachys pubescens* (Li S. et al., 2016), *Phoenix dactylifera* L. (Zouari et al., 2016), *Solanum lycopersicum* (Medyńska-Juraszek et al., 2020), *Triticum aestivum* L. (Lin et al., 2007), *Salvinia auriculata* (Vestena et al., 2011), *Spartina densiflora* (Martínez Domínguez et al., 2010), and *Phyllostachys pubescens* (Li S. et al., 2016). Cd, in bivalent form, is unable to produce free radicals directly; however, after exposure to Cd, there is a significant increase in production of ROS (super oxide radicals, H_2_O_2_, and hydroxyl radicals). Cd induces oxidative stress by counteracting normal antioxidants, i.e., peroxidase (POD), dehydro-ascorbate reductase (DHAR), monodehydroascorbate reductase (MDHAR), ascorbate peroxidase (APX), catalase (CAT), and superoxide dismutase (SOD), and non-enzymatic antioxidants, i.e., vitamins C and E, tocopherols, carotenoids, ascorbic acid (ASA), and glutathione reductase (GR), which results in overproduction of ROS and causes damage to the biosynthetic machinery of cells. This xenobiotic-induced oxidative stress results in damage of biological membranes, macromolecules (proteins, lipids, and phospholipids), and biological membranes of cells. Cd also negatively influences the mitochondrial matrix by disrupting oxidative phosphorylation and ATP synthesis. Exposure to Cd also causes negative impacts on the cell repairing process of enzymatic proteins and damage to DNA and RNA, and reduces the proliferation and differentiation of cells (Akhter et al., 2021).

### Carbon Metabolism and Yield Formation

Plants face toxic environmental constraints and mitigate these by dealing with carbon metabolism, constant supply of CO_2_, maintaining electron transport chain, and assimilation of CO_2_ at certain levels (Leegood, 1993). Disturbance in carbon metabolism occurs because of Cd toxicity and leads to decrease in photosynthetic efficiency (Gouia et al., 2003). Cd effectively causes alteration in photosynthesis by negative influence on its all aspects, including electron transport chain (ETC), photosystems (PSI and PSII), chl-proteins complexes, and CO_2_ reduction pathways in the stroma (Parmar et al., 2013). Cd-induced toxicity also convincingly results in alteration of chloroplast ultrastructure with inflated and disrupted thylakoids (Najeeb et al., 2011). Alteration in chloroplast ultrastructure was manifested by reduction in number of chloroplasts and size and number of grana, accumulation of plastoglobules, and excessive starch in leaves of *Hordeum vulgare, Picris divarticata*, and *Brassica* spp. (Ying et al., 2010; Wang et al., 2011; Elhiti et al., 2012). Moreover, the Willow plant showed aggregation of grana, disrupted thylakoids, and swallowed intrathylakoidal spaces owing to lipid peroxidation (LPX) (Hakmaoui et al., 2007). LPX is ensued by the activity of lipoxygenase (LOX) enzyme (Remans et al., 2010). This enzyme mediates the peroxidation of membrane fatty acids such as phosphatide glycerol (PG) and mono- and digalactosyldiacyl glycerol (MGDG, and DGDG. For instance, more accumulation of the activation of MGDG, DGDG, and PG results in the production of ROS and free radicals. Correlation of LOX with LPX reported in many plants like Lupine, Barely, Phaseolus, and *Arabidopsis thaliana* in response to Cd exposure (Maksymiec and Krupa, 2006; Tamás et al., 2009). In general, Cd causes a significant reduction in carbon metabolism, this reduction causes change in photosynthesis because of low supply of CO_2_, and low carbon levels counteract ETC and thylakoid membranes and photosynthetic enzymes. Ultrastructural changes in cell components like chloroplasts are also a major cause of reduction in photosynthesis efficiency of cells in leaves.

Disruption of photosynthetic pigments and biosynthetic inhibition in old and young leaves of plants have been reported as a primary cause of Cd toxicity (Xue et al., 2013; Anjum et al., 2016). Chlorophyll biosynthesis mainly depends on the aminolevulinate (ALA) compound, and Cd inhibits ALA at the site of glutamate availability and contradicts with the SH functional group of enzyme-like porphobilinogen deaminase and δ-aminolevulinic acid (Myśliwa-Kurdziel and Strzałka, 2002; Skrebsky et al., 2008). However, excessive concentration of ALA is responsible for the production of ROS, which can alter the redox potential of cells and alter cellular homeostatic functions, as reported in *Cucumis sativus* (Goncalves et al., 2009) and soybean (Noriega et al., 2007).

Cadmium toxicity alters the function of photosystems (PSI and PSII), interacts with PSII core complex and PSII supramolecular entities, and retards photoactivation (Quartacci et al., 2000; Sigfridsson et al., 2004). In contrast, PSI is considered more susceptible to Cd toxicity, and it might be because of Cd-induced Fe deficiency, which causes more damage to PSI (Timperio et al., 2007). Iron-deficit damage to PSI has been reported in *Cucumis sativus* L. under 10-μM Cd stress (Sárvári, 2008). In comparison to PSII, PSI is less explored in terms of Cd toxicity; however, photosynthetic yield was severely hindered because of Cd in *Thlaspi caerulescens* and *Pisum sativum* (Küpper et al., 2007; Wodala et al., 2012). Cd toxicity also hampers the Calvin cycle and shows inhibitory effects on various enzyme activities (Ying et al., 2010). Enzymes RUBP and PEP play a vital role during photosynthesis and are involved in CO_2_ fixation (Zhang et al., 2007). Excessive Cd ions decrease the activity of RUBP and PEP by causing alteration in their structures, and replace essential cofactors, such as Mg^2+^, which are involved in the process of carboxylation and shift to oxygenation reactions (Tran and Popova, 2013). Cd stress leads to disruption of photosynthetic traits, which results in damage of chloroplast components and hindrance in vital photosynthetic enzymes (Singh and Prasad, 2017).

Cadmium stress severely hinders plant growth, photosynthesis, and grain yield (Rizwan et al., 2016; Abbas et al., 2018). Several studies reported that Cd translocates to rice grains because of significant decline in grain yield and uptake of nutrients by roots (He et al., 2006; Liu et al., 2007; Rodda et al., 2011; Li and Zhou, 2012). However, in rice, the amplitude of yield reduction depends on genotype, concentration of Cd treatment, and duration of exposure (Song et al., 2017). Cadmium toxicity also severely reduces yield traits like number of spikelets per plant, grain number per ear, ear length, and weight in wheat crop (Khan et al., 2007). For instance, the threshold of Cd toxicity varies from genotype to genotype, exposure duration, and concentration of dose (Rizwan et al., 2016). Reduction in yield of crops has been reported previously on several plant species such as barley (Lentini et al., 2018), pea (Januškaitienė, 2010), tomato (Hayat et al., 2012), *Phaseolus vulgaris* (Rady, 2011), *Zea mays* L. (Anjum et al., 2015), and *Cicer arietinum* L. (Hayat et al., 2013; Liza et al., 2020). Cd toxicity severely reduces the growth and yield traits of plants; however, severity depends on plant species, Cd level, and exposure period. The impact of Cd on different plant crop yields is documented in Table 2.

**TABLE 2:** Impact of Cd toxicity on yield of some representative crops.

Crop species	Level of Cd	Yield reduction (%)	References
Radish (*Raphanus sativus*)	200 mg kg^–1^	29.55–53.48	Varalakshmi and Ganeshamurthy, 2013
Radish (*Raphanus sativus*)	10 mg L^–1^	10.0	Amirabad et al., 2020
Wheat (*Triticum aestivum* L.)	1 mg L^–1^	5.0–9.0	Zhang et al., 2002
Wheat (*Triticum aestivum* L.)	100 mg kg^–1^	26–53	Khan et al., 2007
Rice (*Oryza sativa* L.)	150 mg kg^–1^	38.3–47.1	Huang et al., 2008
Rice (*Oryza sativa* L.)	100 mg kg^–1^	22.16–32	Lin et al., 2016
Rice (*Oryza sativa* L.)	100 mg kg^–1^	15–38	Cao et al., 2015
Cotton (*Gossypium hirsutum* L.)	600 μM	60.6	Li et al., 2012
Mustard (*Brassica juncea* L.)	150 mg kg^–1^	33–79–60	Gill et al., 2011
Canola (*Brassica napus* L.)	12 mg kg^–1^	65.39	Ghani, 2011
Mungbean (*Vigna radiata* L.)	25 mg kg^–1^	26–37	Wahid and Ghani, 2008
Tomato (*Solanum lycopersicum*)	50 μM	25.5	Kumar et al., 2015
Tomato (*Solanum lycopersicum*)	50 μM	10.31–25.50	Xie et al., 2021
Barley (*Hordeum vulgare* L.)	5 μM	10.7–55.8	Wu et al., 2007

## Remediation of Cadmium-Polluted Soils

### Physicochemical Remediation Methods

Remediation of Cd-contaminated soils could be achieved *via* organic chelate and surfactant application, and it is known as chelate-assisted phytoextraction or chelate-induced phytoextraction (Habiba et al., 2015; Sakouhi et al., 2016). Compared to traditional phytoremediation, chelate-induced phytoextraction is more beneficial, convenient, and environment frindly, because chelate-induced phytoextraction augments the extraction aptitude of hyperaccumulators (Clabeaux et al., 2011; Zhao et al., 2015; Wang et al., 2019). In chelate application strategy, different types of amino polycarboxylic acid chelates like *S,S*-ethylenediamine disuccinic acid (EDDS), iminodisuccinic acid (IDSA), [*N, N*]-bis glutamic acid (GLDA), ethylenediaminetetraacetate (EDTA), nitrilotriacetic acid (NTA), diethylenetriaminepentaacetic acid (DTPA), methylglycinediacetic acid (MGDA), ethylenebis (oxyethylenenitrilo) (EGTA) tetraacetic acid, and *trans*-1,2-diaminocyclohexane-*N, N, N0, N0* -tetraacetic acid diethylenetriaminepentaacetic acid (CDTA) are applied in a Cd-contaminated growth medium to escalate Cd mobilization as well as phytoextraction capacity (Table 3; Zaheer et al., 2015; Hasan et al., 2019). Similarly, organic acids having low molecular weight, like oxalic acid (OA), citric acid CA), and tartaric acid (TA), have also been used as chelating agents. Having multi-ligand structures, these chelating agents form stable compounds with HMs and, resultantly, increase the mobility of soil HMs (Guo et al., 2019). According to Bian et al. (2016) and Moslehi et al. (2019), these chelating agents not only enhance the mobility of Cd in the soil solution but also change the form of Cd in soils, boost the availability of Cd for uptake and translocation, and, ultimately, upsurge Cd accumulation in aboveground biomass of plants grown in Cd-contaminated soils.

**TABLE 3:** Chemical remediation of Cd-contaminated soil.

Crop	Cd concentration in soil	Chelate applied	Dose	Cd uptake		References
				Control	Chelate treated	
*Cicer arietinum* L.	200 μM CdCl2.	EGTA	100 μM	300 μg g^–1^	195 μg g^–1^	Sakouhi et al., 2016
*Brassica napus*	0.37 mg kg^–1^		100 kg ha^–1^	0.44 mg kg^–1^	0.33 mg kg^–1^	Bloem et al., 2016
	0.37 mg kg^–1^		500 kg ha^–1^	0.44 mg kg^–1^	0.25 mg kg^–1^	
	0.37 mg kg^–1^		1000 kg ha^–1^	0.44 mg kg^–1^	0.24 mg kg^–1^	
*Neyraudia reynaudiana*	–	EDTA	2.5 m mol kg^–1^	98 mg kg^–1^	184 mg kg^–1^	Li Z. et al., 2018
*Neyraudia reynaudiana*	–	EDTA	5 m mol kg^–1^	98 mg kg^–1^	86 mg kg^–1^	Li Z. et al., 2018
*Amaranthus hybridus* L.	30.15 mg kg^–1^	EDDS	5.0 mmol kg^–1^	99 mg kg^–1^	146 mg kg^–1^	Li Z. et al., 2018
*Amaranthus hypochondriacus* L.	2.12 mg/kg	GLDA	3 mM	15 mg kg^–1^	44 mg kg^–1^	Wang et al., 2019
	2.12 mg/kg	GLDA	5 mM	15 mg kg^–1^	49 mg kg^–1^	
	2.12 mg/kg	NTA	3 mM	15 mg kg^–1^	51.5 mg kg^–1^	
	2.12 mg/kg	NTA	5 mM	15 mg kg^–1^	37.5 mg kg^–1^	
	2.12 mg/kg	CA	3 mM	15 mg kg^–1^	23 mg kg^–1^	
	2.12 mg/kg	CA	5 mM	15 mg kg^–1^	27 mg kg^–1^	
	2.12 mg/kg	EDDS	3 mM	15 mg kg^–1^	44 mg kg^–1^	
	2.12 mg/kg	EDDS	5 mM	15 mg kg^–1^	35 mg kg^–1^	
*Amaranthus hypochondriacus* L.	2.89 mg/kg	GLDA	3 mM	18 mg kg^–1^	36.5 mg kg^–1^	
	2.89 mg/kg	GLDA	5 mM	18 mg kg^–1^	39 mg kg^–1^	
	2.89 mg/kg	NTA	3 mM	18 mg kg^–1^	37.5 mg kg^–1^	
	2.89 mg/kg	NTA	5 mM	18 mg kg^–1^	32.5 mg kg^–1^	
	2.89 mg/kg	CA	3 mM	18 mg kg^–1^	24.5 mg kg^–1^	
	2.89 mg/kg	CA	5 mM	18 mg kg^–1^	26 mg kg^–1^	
	2.89 mg/kg	EDDS	3 mM	18 mg kg^–1^	37 mg kg^–1^	
	2.89 mg/kg	EDDS	5 mM	18 mg kg^–1^	30 mg kg^–1^	
*Helianthus annuus* L.	50 mg kg^_1^	EDDS	5 mmol kg^–1^	1.7 mg pot^–1^	1.6 mg pot^–1^	Moslehi et al., 2019
	100 mg kg^_1^	EDDS	5 mmol kg^–1^	2.9 mg pot^–1^	2.4 mg pot^–1^	
*Tagetes patula* L.	2.44 mg kg^–1^	EDDS	1 mM	832.11 μg/pot	1081.2 μg/pot	
	2.44 mg kg^–1^	EDDS	3 mM	832.11 μg/pot	1088.9 μg/pot	
	2.44 mg kg^–1^	EDDS	5 mM	832.11 μg/pot	619.5 μg/pot	
*Phytolacca americana* L.	2.44 mg kg^–1^	EDDS	1 mM	16.42 μg/pot	72.3 μg/pot	
	2.44 mg kg^–1^	EDDS	3 mM	16.42 μg/pot	144.8 μg/pot	
	2.44 mg kg^–1^	EDDS	5 mM	16.42 μg/pot	64.3 μg/pot	

Among these chelating agents, EDTA has been most widely used because of its slower biodegradability and strong binding affinity toward Cd ions (Chen et al., 2004; Saifullah et al., 2009; Jiang et al., 2019). Structural characteristics of EDTA enable it to form a strong bond with Cd and increase Cd solubilization, translocation, and phytoextraction capacity of phytoremediation (Nowack, 2002; Hasan et al., 2019). However, remediation of Cd-polluted soils varies with different EDTA application rates, plant species, and soil types (Evangelou et al., 2007).

After EDTA, another widely used chelating agent for successful phytoextraction of Cd is EGTA (Pereira et al., 2010). Cheating agent EGTA enhances the uptake of Cd by plants efficiently (Hasan et al., 2019). Previous research studies have highlighted that application of EGTA increased the phytoextraction of Cd by 72% in *Althaea rosea* (Liu et al., 2008) by 43% in *Mirabilis jalapa* (Wang and Liu, 2014), and by 217% in *Calendula officinalis* (Jianv et al., 2010). Similarly, a surfactant named SDS is also being used to remediate organic and metal contaminations in soils (Pierattini et al., 2017). It was concluded that application of SDS not only increased the biomass but also increased Cd accumulation in roots and shoots of *Althaea rosea* (Liu et al., 2009, 2008) and *Calendula officinalis* (Jianv et al., 2010). GLDA is known as a green chelating agent, and it has better Cd extraction efficiency (Wu et al., 2015).

In terms of degradation, EDTA biodegrades slowly, while EDDS is a quick biodegradable chelating agent that can augment the mobility of HMs and their uptake, and translocation and accumulation of HMs in plant shoots in HM-contaminated soils (Li et al., 2013; Attinti et al., 2017; Zhu et al., 2017). However, EDDS phytoextraction efficacy depends on time of application, method of application, dose, and level of Cd contamination in soils.

Similar to EDDS and GLDA, NTA is also a quick biodegradable (approximately 7 days) and highly effective chelate for remediation of Cd-polluted soils (Hu X. et al., 2017). Similarly, application of some other chelating agents like DTPA and IDSA proved to be useful in Cd complexation, enhancing Cd solubility and its uptake by plants, e.g., hydroponically grown maize (Zhao et al., 2010). Saifullah et al. (2009) and Wang et al. (2019) highlighted that besides application of single chelating agents, when chelator complexes (a combination of two different chelates), e.g., GLDA + NTA and GLDA + CA were applied on Cd-contaminated soils, biomass production and Cd uptake by plants increased significantly.

Contrarily, where chemical amendments using chelating agents are conducted to phytoextract Cd from soil (Lambrechts et al., 2011), at the same time, these chemical amendments have a limitation of stunted plant growth, e.g., *Pseudomonas brassicacearum* (Krujatz, 2012), *Lolium perenne*, *Brassica juncea*, and *Typha angustifolia* (Muhammad et al., 2009; Goel and Gautam, 2010; Xu et al., 2010). Under such circumstances where the application of a single chelating agent resulted in stunted growth, application of a quick biodegradable chelate complex is a potential option for enhancing Cd phytoextraction (Wang et al., 2019). Similarly, combined application of chelating agents and plant growth regulators increased Cd uptake and biomass accumulation (Li Z. et al., 2018). Hence, in crux use of chelating agents is a viable option for remediation of Cd polluted soils. The application of chelating agents significantly enriched Cd uptake in plant biomass of many important plants (Table 3). Studies propose that application of chelates is a viable strategy for remediation of Cd-polluted soils. Soil contamination with heavy metals is a widespread environmental constraint. Therefore, it is very important to reduce the toxic impacts of HMs and their associated risks to plants and restoration of soils. The phytoremediation process includes phytostimulation, phytofiltration, phytotransformation, and phytoaccumulation, which extensively reduced the noxious effects of HMs in soils. The soil physicochemical remediation process includes soil washing, vitrification, solidification, stabilization, and use of metallophytes for phytoextraction. The process of phytoremediation of HM-contaminated soils is a reliable tool and necessary to make land resources accessible for crop production.

### Plant Growth Regulators Assisted Remediation

Modulation of phytohormones or plant growth regulators (PGRs) not only mitigates the toxic effect of Cd stress on plants (Li Y. et al., 2018) but also enhances the tolerance of plants to Cd stress (Asgher et al., 2015) and efficacy of phytoextraction of Cd by plants in Cd-polluted soils (Sun et al., 2020). Research studies suggested the positive effect of PGRs on Cd translocation and accumulation, promotion of plant growth and nutritive value, and biomass accumulation under Cd stress (Asgher et al., 2015; Aderholt et al., 2017; Chen et al., 2019). Among key PGRs, cytokinins (CKs), ethylene, gibberellins (GAs), auxins, abscisic acid (ABA), polyamines (Pas), jasmonic acid (JA), brassinosteroids (BRs), and nitric oxide (NO) play substantial roles specifically in plant growth and developmental processes (Asgher et al., 2015). According to Piotrowska-Niczyporuk et al. (2012) and Sun et al. (2020), exogenous application of PGRs acts in diverse modes and enhances plant adaptability and tolerance to Cd stress in different ways. In addition, the role of CKs, indole-3-acetic acid (IAA), indole-3-butyric acid (IBA), and 1-naphthaleanecetic acid (NAA) in phytoextraction of Cd from Cd-polluted soils has been documented in many studies (Table 4; Bulak et al., 2014; Okem et al., 2015).

**TABLE 4:** Plant growth regulators assisted remediation of Cd-polluted soils.

Crop	Cd concentration in soil	PGR	Dose	Effect		References
				Control	PGR treated	
*Vicia faba*	150 mg L^–1^	Jasmonic acid	0.01 mM	43.2 μmol g^–1^	17.3 μmol g^–1^	Ahmad et al., 2017
*Mentha piperita* L.	30 mg kg^–1^	Salicylic acid	10 μM	6 μmol g^–1^	7 μmol g^–1^	Ahmad et al., 2018
*Mentha piperita* L.	60 mg kg^–1^	Salicylic acid	10 μM	6 μmol g^–1^	14 μmol g^–1^	
*Mentha piperita* L.	120 mg kg^–1^	Salicylic acid	10 μM	6 μmol g^–1^	34 μmol g^–1^	
*Zea mays L.*	0.5 mM	Salicylic acid	0.5 mM	46.3 μmol g^–1^	14.7 μmol g^–1^	Gondor et al., 2016
*Oryza sativa*	50 μM	Salicylic acid	50 μM	166.7 μg g^–1^	90.6 μg g^–1^	Singh et al., 2018
*Brassica napus* (Zheshuang-72)	75.12 mg kg^–1^	Salicylic acid	50 μM	125 mg g^–1^	80 mg g^–1^	Ali et al., 2015
	150.12 mg kg^–1^	Salicylic acid	50 μM	185 mg g^–1^	125 mg g^–1^	
	300.12 mg kg^–1^	Salicylic acid	50 μM	240 mg g^–1^	175 mg g^–1^	
*Brassica juncea* L.	15.31 mg kg^_1^	Indole acetic acid	100 mg L^–1^	98.1 mg g^–1^	95.3 mg g^–1^	Chen et al., 2020
	15.31 mg kg^_1^		250 mg L^–1^	98.1 mg g^–1^	107.8 mg g^–1^	
	15.31 mg kg^_1^		500 mg L^–1^	98.1 mg g^–1^	138.5 mg g^–1^	
*Brassica juncea* L.	15.31 mg kg^_1^	Gibberellic acid	100 mg L^–1^	98.1 mg g^–1^	99.8 mg g^–1^	Chen et al., 2020
	15.31 mg kg^_1^		250 mg L^–1^	98.1 mg g^–1^	115.2 mg g^–1^	
	15.31 mg kg^_1^		500 mg L^–1^	98.1 mg g^–1^	95.3 mg g^–1^	
*Brassica juncea* L.	15.31 mg kg^_1^	24-Epibrassinolide	5 mg L^–1^	98.1 mg g^–1^	96.3 mg g^–1^	Chen et al., 2020
	15.31 mg kg^_1^		10 mg L^–1^	98.1 mg g^–1^	120.5 mg g^–1^	
	15.31 mg kg^_1^		50 mg L^–1^	98.1 mg g^–1^	132.5 mg g^–1^	
*Amaranthus hybridus* L.	30.15 mg kg^–1^	Diethyl aminoethyl hexanoate	10 μM	99 mg kg^–1^	125.7 mg kg^–1^	Li Y. et al., 2018
	30.15 mg kg^–1^		100 μM	99 mg kg^–1^	113.5 mg kg^–1^	
*Amaranthus hybridus* L.	30.15 mg kg^–1^	6-Benzylaminopurine	10 μM	99 mg kg^–1^	110 mg kg^–1^	Li Y. et al., 2018
	30.15 mg kg^–1^		100 μM	99 mg kg^–1^	100 mg kg^–1^	

#### Gibberellins

Gibberellins (GA) can also protect plants from negative impacts of trace metals by reducing oxidative stress and increasing antioxidant mechanisms (Nguyen et al., 2020). GAs enhance sulfate assimilation, which promotes GSH/phytochelatin production, as S-containing metabolites are important for plant defense mechanisms. Application of GAs increased biomass accumulation, Cd uptake efficacy of *Lolium perenne* L. (He et al., 2014) and *Helianthus annuus* L. (Long et al., 2017), hampered MDA contents and oxidative stress of *B. juncea* under Cd toxicity (Meng et al., 2019), and in lupin plants and broad beans, it mitigated Cd toxicity by increasing soluble proteins under Cd stress (Sharaf et al., 2009). GAST1, a GA-stimulated transcript implicated in the control of ROS buildup, was also upregulated after an exogenous gibberellin was applied and subsequent transcriptomic techniques were employed (Sun et al., 2013). GA signaling boosted the expression of adenosine 50-phosphosulfate reductase, an enzyme crucial in sulfate assimilation, in *A. thaliana* under stress (Koprivova et al., 2008).

#### Abscisic Acid

Abscisic acid (ABA) is a plant hormone that regulates many aspects of plant development, growth, and stress responses (Nian et al., 2021). Reduced seed dormancy and wilty phenotypes are seen in ABA-deficient mutants from a variety of plant species, indicating that these important ABA activities are maintained across the plant kingdom (Haider et al., 2021b). In *Bidens pilosa*, the application of a stress hormone, e.g., ABA, enhanced plant tolerance to Cd stress and Cd extraction from Cd polluted-soil (Liu et al., 2017; Pompeu et al., 2017). Li et al. (2014) concluded that pretreatment of ABA decreased the activities of ascorbic acid, CAT, SOD, APX, POD, and GSH in roots of *Vigna radiate* L. under Cd stress. ABA, when used as a pretreatment before Cd treatment, did not result in increased contents of cysteine (CYS) and phytochelation (PC). This suggests the role of ABA in the regulation of PCS (Stroiński et al., 2013). The protective role of ABA against Cd stress has also been demonstrated by experiments comparing wild-type *Arabidopsis* plants and ABA-deficient plants, in which the mutants proved to be more sensitive to Cd metal stress (Sharma and Kumar, 2002). These findings strongly suggest that ABA may be involved in signal pathways during Cd stress.

#### Nitric Oxide

Nitric oxide is a free radical that reacts with oxygen molecules and controls the deposition of oxygen in plant tissues (Tran et al., 2011). NO is a signal molecule that activates cell defense mechanisms in response to a variety of stressors (Tran and Popova, 2013). Tran and Popova (2013) and Xu et al. (2015) documented that NO application diminished the structural modification of leaves, increased nutritional value, and improved antioxidant enzyme activities under Cd Stress. In other studies, application of NO on *Cucumis sativus* L. under Cd stress augmented chlorophyll contents and biomass accumulation, and decreased chlorosis symptoms and oxidative stress in plants (Yu et al., 2013).

#### Salicylic Acid

An endogenous phenolic PGR such as salicylic acid (SA) governs an imperative role in plant physiological processes (photosynthesis, growth, and development), specifically under abiotic stresses, including Cd toxicity (Tran and Popova, 2013; Gruznova et al., 2018). Ahmad et al. (2011) highlighted that SA in initial growth stages assisted plants in mitigating increased damage caused by Cd toxicity by expressing specific proteins and defense-related enzymes (Çanakci and Dursun, 2012; Roychoudhury et al., 2016). Pretreatment of SA abridged Cd accumulation, electrolyte leakage, and level of MDA in wheat shoots under Cd stress (Shakirova et al., 2016), enriched the level of lipids, upregulated the antioxidant system, and caused variations in fatty acid composition of vegetable seedlings (Tran and Popova, 2013; Semida et al., 2015; Rizwan et al., 2017). Increased levels of SA, in response to Cd stress, are reported in pea (Rodríguez-Serrano et al., 2006), maize (Krantev et al., 2008), *Arabidopsis* (Zawoznik et al., 2007), and halophyte *Kosteletzkya virginica* (Han et al., 2013). The role of SA in modulating the oxidative stress caused by Cd toxicity is evident by comparing the SA accumulating and deficient lines of Arabidopsis (Zawoznik et al., 2007; Tao et al., 2013). Mutants showed variable levels of H_2_O_2_ contents, lipid peroxidation, and antioxidant enzymes in comparison with wild plants. Increased levels of endogenous SA led to growth retardation while mutants having decreased endogenous SA showed least retardation in growth because of Cd stress. However, majority of studies disclose the protective role of SA in reduction of oxidative stress caused by Cd.

#### Jasmonic Acid

Jasmonates are oxylipins, which are oxygenated fatty acid derivatives (Ahmad et al., 2017). Methyl JA (MeJA) is a volatile molecule that may have a role in plant-to-plant communication. Exogenous application jasmonic acid (JA) resulted in reduced Cd, H_2_O_2_, and malondialdehyde accumulation in *Vicia faba* L. (Ahmad et al., 2017). Similarly, exogenous application of GB reduced the oxidative stress caused by Cd stress and increased the biomass of wheat (Rasheed et al., 2014). Application of ethylene on *Arabidopsis thaliana* under Cd stress increased root proliferation by modulating superoxide anion (Abozeid et al., 2017). At low levels (10−4 mol/L), it may incur a protective role in mitigation of Cd stress, but at high concentrations, JA might induce retardation in growth by degrading chlorophyll and photosynthetic enzymes (Maksymiec and Krupa, 2002).

#### Auxins

The auxin IAA is a well characterized hormone and is involved in growth regulation and physiological development of plant; however, it is still a less studied and less understood mystery in terms of response to Cd stress (Farooq et al., 2015; Teiri et al., 2018). Exogenous application of IAA on *B. Juncea* promoted uptake and accumulation of Cd that might be attributed to increased cell division, formation of vascular tissue and development of a broader root system that as a result, reduces the toxic effect of Cd (Teiri et al., 2018; Rostami and Azhdarpoor, 2019). Farooq et al. (2015) opined augmented growth and yield of rice when a precursor of an auxin was applied in Cd-contaminated soil. Elobeid et al. (2012), in their experiment, reported disturbed homeostasis of auxin in response to exogenously applied Cd. Yu et al. (2017) stated that an auxin transporter (OsAUX1) induced the extension of root hair and primary roots of rice under Cd stress. Ostrowski et al. (2016) reported that the application of an auxin conjugate (IAA-Asp) induced reduction of H_2_O_2_, and upregulated POD and CAT activity under Cd stress.

#### Cytokinins

Cytokinins (CKs) are a kind of plant hormone that promotes cytokinesis (cell division) in plant roots and shoots (Singh and Prasad, 2016). They have a role in cell proliferation and differentiation, as well as apical dominance, axillary bud development, and leaf senescence. Exogenous application of CKs inhibited Cd biosorption and augmented the activities of antioxidant enzymes in *Chlorella vulgaris* (Piotrowska-Niczyporuk et al., 2012) and tomato under Cd-contaminated soil conditions (Singh and Prasad, 2016).

#### Brassinosteroids

Brassinosteroids (BRs) are endogenous plant hormones that regulate a variety of physiological processes that are necessary for appropriate plant growth and development (Rehman et al., 2022a,b). Application of 28-homobrassinolide (homoBL) on the foliage of *Brassica juncea* improved Cd tolerance because of enhanced activity of antioxidant enzymes (CAT, POD, and SOD) (Hayat et al., 2007). The effect of 24-epibrassinolide (24-epiBL) was examined on *Phaseolus vulgaris* in response to Cd stress (Shahzad et al., 2018). 24-epiBL improved membrane stability index, proline content, and antioxidant system (Rady, 2011). Plants exposed to Cd stress have impaired electron transport (ETC) because of demolished photochemical reaction centers, whereas epi-brassinolide (EBL) reduced Cd toxicity and impaired the reaction centers of photosystems and ETC (Janeczko et al., 2005). Moreover, Cd stress helped older tissues more effectively and EBL improved the photosynthetic activity in radish leaves (Anuradha and Rao, 2009). Similarly, in tomatoes, BR application reduced the phytotoxic effects of Cd and improved fruit quality and yield (Hayat et al., 2012). Application of EBL in bean plants subjected to Cd stress increased the levels of antioxidant enzymes. Rise in an antioxidant system (superoxide dismutase, catalase, peroxidase and glutathione reductase, and proline) leads to increased tolerance, enhanced photosynthetic machinery, and growth. Similar kind of results were reported on other crops, i.e., mustard (Hayat et al., 2007) and chickpea (Rady, 2011), by exogenous application of EBL and HBL, respectively. The application of BRs as a shotgun approach (EBL and HBL) improved the chlorophyll content and photosynthesis efficiency of Cd- stressed tomato plants (Hayat et al., 2010). Besides this, BR treatment significantly increased the number of fruits, fruit yield, lycopene, and β-carotene contents in fruits of plants grown under Cd stress.

#### Polyamines

Polyamines are necessary for cell growth. Polyamine content is greater in quickly expanding tissues, and growth-promoting and regenerative hormone cues boost polyamine production and content (Hasanuzzaman et al., 2019). Rady and Hemida (2015) discussed that presoaking wheat seeds with polyamines, spermine or spermidine resulted in enhanced seedling growth, relative water contents, starch, ascorbic acid, membrane stability index, total glutathione, and concentration of protein, and that H_2_O_2_, total soluble sugars, concentration of proline, electrolyte leakage and MDA were decreased under Cd stress.

Some other compounds (paclobutrazol, daminozide, humic acid, and melatonin) are extensively used worldwide to decrease the devastating effects of Cd stress in cultivable plants (Khan et al., 2017; Hasan et al., 2019). In conclusion, the use of the above-mentioned PGRs might be an effective, eco-friendly strategy to enhance the growth and development of plants cultivated in a Cd-stressed environment.

### Microbe-Assisted Remediation of Cadmium Stress

Soil microorganisms may not destroy or degrade HMs; however, they can affect physical and chemical characteristics that might help to migrate and transform them from highly toxic to less toxic forms and restrict their uptake by plants through a number of mechanisms including extracellular complexation, intracellular accumulation, and oxidation–reduction reaction. Furthermore, soil microorganisms could improve plant health by improving the uptake of nutrients that could upregulate plant growth and biomass production (Nejad et al., 2017; Rizwan et al., 2017). Thus, microbial symbioses have imperative ecological roles and can be used to increase the resilience and sustainability of ecosystems (French, 2017), especially in Cd-contaminated areas. Cd-induced polluted soil remediation can be mediated by two common types of mycorrhizae: (1) Ecto-mycorrhiza (ECM) and (2) Arbuscular mycorrhiza (AM).

In all ecosystems, AM and the other fungus, ECM, have made associations with almost all plant species (Lehmann et al., 2017). AM is a unique one, as it colonizes almost all types of plants to remediate HMs; however, unlike AM, ECM mostly colonizes woody plants. Phytoextraction of Cd mediated by *Phragmites australis* was observed under low Cd stress, and immobilization of Cd has in roots under high Cd stress has been reported (Huang et al., 2017). Sell et al. (2005) inoculated *Populus canadensis* and *Salix viminalis* with ECM strains including *Hebeloma crustuliniforme*, *Pisolithus tinctorius*, and *Paxillus involutus*, and observed an increase in Cd uptake and translocation from a sterilized Cd-contaminated soil. Hence, it can be stated that along with ECM inoculation, choice of the host plant is a determinant of better results. Arbuscular mycorrhizal fungi (AMFs) form a mycorrhizal symbiosis with almost 80% of higher plants and may increase the remediation of Cd-contaminated soils (Abdel-Latef et al., 2016; Table 5). The increasing attention on AMFs’ aptitude to retain HMs in the mycelium is due to a process known as “mycorrhizal-remediation,” which decreases the translocation of metals to other plant parts such as shoots and subsequently increases plant tolerance under such conditions (Moreira et al., 2015).

**TABLE 5:** Bioremediation potential of arbuscular mycorrhizae fungi (AMF) against Cd toxicity.

Mycorrhizae	Plant	Mechanisms of heavy metals alleviation	References
*Aspergillus aculeatus*	*Cynodondactylon* (L.)	Alteration of metabolites, IAA production, and Higher relative growth rate (RGR) and normalized relative transpiration rate (NRT).	Li et al., 2017a
*Alternaria alternata*	*Solanum nigrum*	High antioxidant activity, Improvement in plant photosynthetic efficiency, Attenuated lipid peroxidation	Li et al., 2017b
*Glomus versiforme*, *Funneliformis mosseae*, *Rhizophagus intraradices*	*Zea mays* L.	This study demonstrated a synergistic effect between AMF and biochar on improving maize growth and decreasing Cd/Pb accumulation in maize	Zhuo et al., 2020
*Glomus intraradices*	*Zea mays* L. *Zea mays L.*	Decreasing Cd phyto-toxicity due to the synergistic effect of microbes and biochar	
*Rhizophagus intraradices, Glomus versiforme*	*Lonicera japonica*	Microbial symbiosis ameliorated the Cd toxicity by reducing Cd content in shoot and improved of P uptake	Liu et al., 2018
*Glomus versiforme Glomus elunicatum*, *Glomus aggregatum*, *Glomus intraradices*,	*Medicago sativa*	Reduced Cd content	
*Rhizophagus irregularis*	*Glycine max*	Arbuscular mycorrhizal colonization had no impact on Cd concentration and translocation in HN89 and HX3 plants	Jiang et al., 2016
*Glomus aggregatum Rhizophagus fasciculatus, Funneliformis mosseae, Rhizophagus intraradices*	*Zea mays*	Phytoextraction	
*Glomus geosporum Glomus mosseae Glomus intraradices Glomus claroideum*	*Nicotiana tabacum*	Reduced the Cd mobility in the soil (Phytostabilization)	Zhang et al., 2019
*Glomus intraradices*	*Zea mays*	Cd concentration was reduced by improving the growth of maize; Sequestered Cd toxicity by upregulating the activities of SOD, POD, and CAT	
*Scutellospora* sp. *Gigaspora* sp. *Acaulospora* sp. *Glomus* sp.	*Fabaceae, Asteraceae, Poaceae*	Improved glomalin protein to sequester Cd content	Cui et al., 2019
*Glomus* sp.	*Triticum aestivum*	Phytostabilization potential to sequester Cd; Cd immobilization	
*Rhizophagus irregularis*	*Phragmites australis*	Cd toxicity ameliorated by improving photosynthesis rate, root biomass, micro- and macro-element concentrations in plants and decreased the malonaldehyde (MDA) and proline content; Reduced stomatal conductance and transpiration rate to alleviate Cd toxicity	Singh et al., 2019
*Rhizophagus intraradices G. versiforme*	*Lonicera japonica*	Decreased MDA by improving P acquisition, antioxidant activity (CAT, APX, and GR)	
*G. mosseae*	*Apium graveolens*	Increased chlorophyll content, P accumulation, and plant growth by increasing phytoextraction in Cd stress	Janoušková et al., 2006
*G. mosseae*, *G. intraradices*, *G. etunicatum*	*Cassia italic* Mill	Cd stress mitigated by the enhanced production of antioxidants, chlorophyll, and protein content, and osmoprotectants including proline and phenol content	

Inoculation of AMF improved the metal content in organic matter, electrical conductivity, soil pH, and the proportion of bioavailable Cd in a post-harvest soil (Wu et al., 2016). Nevertheless, *Glomus versiforme* significantly increased the translocation of Cd from roots to shoots relative to *Fusarium caledonium*. Furthermore, HMs cause oxidative stress in plants because of overproduction of ROS normally occurring during plant metabolism. There is always a balance between production and utilization of ROS in plant cells (Riaz et al., 2018; Yan et al., 2018; Kamran et al., 2019). AMF (*Rhizoglomus intraradices*, *Glomus etunicatum*, and *Glomus versiforme*) inoculation upregulated the activity of antioxidant enzymes in Cd-stressed plants that helped to increase plant growth and biomass (Tanwar et al., 2015; Sharma et al., 2017; Liu et al., 2018; Molina et al., 2020). Synthesis of sulfur-rich compounds such as phytochelatins and glutathione is important to mediate plant tolerance among numerous stress-induced detoxification pathways activated in plants (Mishra et al., 2009). AM’s role in regulation of thiol metabolism has recently been associated with Cd stress (Garg and Chandel, 2015). Thus, the defensive effect of AM is embodied in the mediation of the antioxidant enzyme system, alleviating the index of the lipid peroxidation process (Zhan et al., 2018; Janeeshma and Puthur, 2020; Luo et al., 2020), and AM inoculation improved phenol contents and proline and reduced H_2_O_2_ and lipid peroxidation (Hashem et al., 2016). Mycorrhizal plants show greater tolerance against metal stress through mechanisms such as chelation of metals in hyphae, immobilization, glomalin, root colonization, and compartmentalization in fungal cells (Yang et al., 2017). Inoculation of *Anaerolineaceae* in bioremediation of Cd stress is another possible method, because this regulates the shaping of microbial communities and mediates Cd solubility. AM (*Rhodobacter sphaeroides*) helped in P solubilization (Chen et al., 2019), Fe nutrition (Lehmann and Rillig, 2015), P and N uptake under different irrigation regimes (Liu et al., 2018), and enhanced the levels of K, P, and Ca in *Euonymus japonica*, and prominent levels of Zn, Mn, P, and K were sustained under stress (Bagheri et al., 2012). Chang et al. (2018) studied the putative role of *Claroideoglomus etunicatum* fungus that improved the uptake of N, P, and K by 20.1–76.8%, and by decreasing Cd uptake. AMF-induced glomalin accumulation acts as a defense system in plants against Cd-mediated oxidative stress in soils and plant tissues (Babadi et al., 2019). Bioaugmentation is a method that could be beneficial under low native AMF inoculum potential and involves the addition of microbial population for remediation of contaminated areas (Mongkhonsin et al., 2019). New stimulant formulations and techniques aimed at producing AMF inoculants might instigate the prevalent practice of AMF inoculation in the near future. Microbial flora such as fungi, algae, and photosynthetic flora effectively reduce HM contamination. Microbes showed various possible mechanisms to eliminate Cd toxicity including sequestering or accumulating metals in their cell walls and altering the composition of toxic compounds. Cadmium can be introduced to bacterial cells in the form of divalent cations by gene amplification, active efflux, and active influence on metallothionein genes. The major potential of remediation of metals by microbes is low operating costs, high capacity, metal recovery potential, and effective biosorbent regeneration.

#### Plant Growth-Promoting Rhizobacteria

**FIGURE 4:**
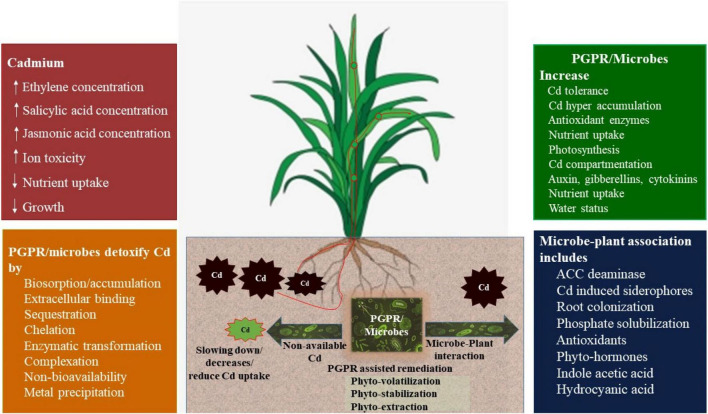
Impact of plant growth-promoting rhizobacteria (PGPRs) on remediation of Cd-contaminated soils (conceived from Ullah et al., 2015; Sharma and Archana, 2016).

Plant growth-promoting rhizobacteria (PGPRs) are rhizosphere inhabitants that enhance plant growth by improving plant nutrient availability, water relationship, and antioxidant activity to improve abiotic stress tolerance (Saeed et al., 2021). They are categorized based on (a) inherent characters (Kloepper, 1994): they (i) enhance root colonization, (ii) improve plant growth, (iii) acclimatize, survive, reproduce, and compete until expression of their potential in plant growth promotion/protection; (b) functional properties (Ahemad, 2014): (i) phytostimulators (phytohormones improve growth), (ii) biofertilizers (regulate nutrient uptake), (iii) rhizoremediators (solubilization of metals), and (iv) biopesticides (regulate plant diseases and pathogens by producing metabolic compounds and lytic enzymes). PGPRs help in phytoremediation; production of soluble minerals, siderophore, phytohormones, rhamnolipid, extracellular polymeric substances, osmo-protectants, 1-aminocyclopropane-1-carboxylate deaminase (ACCD), immobilization of metals (Mahajan and Kaushal, 2018), bioremediation; accumulation or transformation of contaminants, rhizoremediation; and remediation of contaminated soils by symbiotic relationship between plant roots and suitable microbial species. Inoculation of PGPRs has been reported to decrease Cd uptake and alleviate Cd-induced oxidative stress by producing phytohormones (Glick, 2014; Wang et al., 2015), ammonia, and siderophores that mediate nutrient availability, plant biomass accumulation, and plant water status (Ahmad et al., 2015; Hassan et al., 2015; Figure 4). However, Cd uptake may be improved by inoculation of Cd-tolerant bacteria in plants (Bojorquez et al., 2016; Sharma and Archana, 2016), which depicts the specificity of PGPRs and aim of experiment, i.e., phytostabilization vs. phytoextraction. Under heavy metal stress conditions, PGPR-induced IAA acts as a phytohormone to improve cell division and elongation to stimulate root growth, and enhances root nodulation, vascular bundle development, and plant growth (Goswami et al., 2016; Chen et al., 2017). *Pseudomonas aeruginosa* strains ZGKD5 and ZGKD2 augment to synthesize IAA in *Solanum nigrum* to improve tolerance against Cd stress (Shi et al., 2016). Huang et al. (2016) observed that Pse-w-MT induces Cd tolerance in *Pisum sativum* L. by improving the production of IAA. A *Bacillus megaterium* strain regulated cytokinin production by mediating the transcriptional level of the roots and shoots s receptor (AHK3/AHK4) that induced root morphogenesis in *Arabidopsis thaliana* (Jianfeng et al., 2017). Cd toxicity and uptake have been decreased by inoculation of Cd-resistant *Micrococcus* sp. TISTR2221 in maize (Suksabye et al., 2016) and *Pseudomonas aeruginosa* and *Bacillus subtilis* in rice (Sangthong et al., 2016).

Extracellular polymeric substances (EPSs), mucopolysaccharides, and proteins are produced by different PGPRs that help to bind toxic HMs for thriving plant growth (Upadhyay et al., 2011; Rajkumar et al., 2012). PGPR strains have been identified that produce EPS to counter the toxic effect of Cd contamination by decreasing the uptake of Cd in wheat (Joshi and Juwarkar, 2009). *Pseudomonas aeruginosa* inoculation with a PGPR strain helps to detoxify Cd stress based on extracellular biosorption, bioaccumulation, controlled siderophore production, formation of biofilm, enhanced respiration, and modified protein profile (Zivkovic et al., 2018). Recent studies have also shown the detoxification of Cd stress by *P. aeruginosa* (Zivkovic et al., 2018) and its adapted cells (Tang et al., 2018). Cd stress involved in the uptake of different nutrients influences metal solubility, and P solubilization, and improves nutrient mobility (chelation, precipitation, and immobilization) (Rafique et al., 2017; Saeid et al., 2018). PGPRs could be a promising option to enhance phyto-stabilization efficiency and bioleaching of Cu and Cd in heavily polluted soils owing to biosorption or bioaccumulation (Yang et al., 2018; Ke et al., 2021). Plants produce ROS-scavenging enzymes to detoxify ROS, which are produced under HM stress (Zainab et al., 2020). Exogenous applications of PGPR a strain, *Streptomyces* strain IT25, decreased catalase (CAT),and it is reported that PGPRs upregulated SOD (superoxide dismutase), POD (peroxidase), and PPO (plant polyphenol oxidases) genes to sequester Cd stress (Khanna et al., 2019; Abbasi et al., 2020) (Table 6). *Azospirillum brasilense* may ameliorate the negative effects of Cd stress by upregulation of lower Na/K ratio, *TaSOS1* transcript level, proline, higher pigments, and antioxidant activities to improve dry weight (Ghassemi and Mostajeran, 2018). PGPR-mediated remediation of HMs and promotion of plant growth depend on PGPR strains coded by several genes and must be understood to build a multidimensional PGPR strain to perform multidimensional functions. Nevertheless, use of genetically engineered PGPR strains with hyper-accumulator plants to remove HMs is less common (Ullah et al., 2015). Several studies have also documented that genetic engineering mediated the improvement in remediation of HM stress in plants (Verma et al., 2017). Qiu et al. (2014) incorporated a bifunctional glutathione synthase gene (gcsgs) into *Enterobacter* sp. CBSB1 improved the efficiency of HM removal in *B. juncea*.

**TABLE 6:** Influence of inoculation with plant growth-promoting rhizobacteria (PGPRs) on Cd chelator and transporter gene expression (MT: metal transporter 1).

Conditions	Plant	Genes	PGPR	Mechanisms	References
Hydroponic media	*Triticum aestivum*	Tatm20	*Azospirillum brasilense*	Cd tolerance	Ghassemi and Mostajeran, 2018
Hydroponics	*Sarcosphaera coronaria*	PCS, F-box	PGPR strains	Improved Cd tolerance and Cd transport	Jebara et al., 2018
Hydroponic	*Sedum alfredii*	NRAMP, HMA family genes, ZIP	*Endophytic bacterium*	Improved C uptake, Regulated plant acquisition of essential nutrients	Pan et al., 2017
Hydroponic	*Sedum alfredii*	NRAMP, HMA family genes, ZIP	*Pseudomonas fluorescens*	Cd uptake	Chen et al., 2017
Growth chamber	*Arabidopsis thaliana*	IRT1, FIT1, FRO2	*Bacillus amyloliquefaciens*	Enhancing uptake	Zhou et al., 2017
Controlled conditions	*Lycopersicon esculentum*	6MT, MT3, 5, MT1, MT7	*Burkholderia gladioli* and *Pseudomonas aeruginosa*	Enhancing photosynthetic pigments and growth	Khanna et al., 2019
Soil less pot s system	*Cucumis melo* L.	Fe (IRT1, FRO), NH^4+^ (AMT2), Pi (PHT1)	*Enterobacter asburiae*, *Pseudomonas koreensis*, *Pseudomonas lini*	Enhanced the yield of fruit	Murgese et al., 2020
Green house experiment	*Medicago sativa*	*NRAMP1*	*Proteus* sp., *Pseudomonas* sp., *Ensifer meliloti*	Remediate metal-contaminated soils	Raklami et al., 2019

### Cadmium Remediation Through Organic Amendments

To minimize the noxious effects of Cd in plants, use of soil additives is a promising method to fix Cd in soils because of its *in situ* assistance, low cost, and low energy needs (Yao et al., 2021). However, immobilization of Cd in soils has been performed using different inorganic and organic amendments. Various chemical and biological reactions are involved in Cd immobilization through these amendments (He et al., 2019). Two major sources of organic amendments are plants and animals that can increase soil fertility, as they are saturated with carbon, hydrogen, and oxygen (Hamid et al., 2020a). Use of organic amendments in agricultural soils have many benefits, but their most promising role is immobilization (Hu W. et al., 2017). Biochar, compost, and manure have been used as chief organic amendments for remediation of soil Cd (Hamid et al., 2019) through chelation, adsorption, and precipitation (Hamid et al., 2020a; Mondal et al., 2020).

#### Biochar

To overcome the deleterious effects of HMs, biochar addition to soil is a practical approach (Yuan et al., 2019). Soil productivity and growth of plants have been increased with biochar (Qayyum et al., 2017). In the past years, biochar (BC) has been used in HM-contaminated soils because of its higher adsorption capacity and ability to lower HM contents in soils (Boostani et al., 2019). Use of biochar is an efficient approach to minimize HMs in soils, but it is expensive compared to other amendments in terms of production (Sohail et al., 2020). Biochar, an organic soil additive, has been very helpful in immobilizing the in soils (Hamid et al., 2020b; Haider et al., 2022). Its role in Cd immobilization is auspicious because of its basic nature, porous texture, energetic functional groups, and higher CEC (Medynska-Juraszek and Cwielag-Piasecka, 2020). Biochar-amended soils showed lower Cd transport and accumulation (Abbas et al., 2018). Biochar can easily adsorb Cd, Pb, and Cu by forming complexes and cation exchange mechanisms in soils (Farooq et al., 2020). Breakdown of natural organic material under controlled temperature and limited or no oxygen resulted in biochar production (Hussain et al., 2021). It is well-reported in the published literature that the use of biochar in pots and field tests has significantly improved growth, biomass, and economic productivity in Cd-contaminated soils (Table 7).

**TABLE 7:** Effect of biochar on remediation of Cd-contaminated soils.

Plant species	Feed stock	Applied rate	Experiment type	Soil type	Heavy metals	Effects	References
Rice (*Oryza sativa*)	Rice Straw (450°C)	0, 3, and 5% (w/w)	Pot	Sandy clay loam	Cd	Biochar application significantly decreased the Cd uptake (38%) along with a considerable increase in plant growth.	Hafeez et al., 2019
Pak choi (*Brassica chinensis*)	Rice straw (550°C)	0, 2.5 and 5% (w/w)	Pot	Alfisol	Cd	Application of biochar reduced the Cd uptake in root (29.23%) and shoot (42.49%), while increased the plant production together with enhanced enzymatic antioxidant activity.	Kamran et al., 2019
Saffron (*Crocus sativus*)	Beeswax waste (400°C)	0, 1.5, 3 and 6% (w/w)	Pot		Cd	The Cd uptake was reduced up to 24% in corm and 33% in leaf coupled with increased plant biomass with biochar application.	Moradi et al., 2019
Spinach (*Spinacia oleracea*)	Cotton stalk, Rice straw (450°C)	0, 2 and 5% (w/w)	Field		Cd	Both the biochar applications minimize the Cd uptake in plants up to 66% and enhanced the fresh biomass of spinach and phosphorous concentration in the soil.	Qayyum et al., 2019
Spinach (*Spinacia oleracea*)	Cotton stalk, Rice straw (450°C)	0 and 2% (w/w)	Pot		Cd	The treatments of both rice and cotton biochar considerably increased the fresh mass and reduced the Cd uptake (61%).	Qayyum et al., 2019
Maize (*Zea mays*)	Common reed (550°C)	0 and 1% (w/w)	Pot	Alkaline soil	Cd	Application of biochar enhanced the plant biomass, root length, and root volume in addition to reduced Cd uptake (57%).	Rafique et al., 2019
Pak choi (*Brassica chinensis*)	*Platanus orientalis* branches (650°C)	0, 0.5, 1, 2, and 4% (w/w)	Pot	Loamy soil	Cd	Biochar application reduced the Cd availability (80%) and malondialdehyde concentration in the shoot.	Chen et al., 2019
Garden lettuce (*Lactuca sativa*)	Rice husk (500°C)	0 and 5% (w/w)	Pot		Cd, Pb, As, Ni, Cr	Application of biochar reduced the bioavailability of Cd (31%), Pb (20%), and As (22%) in addition to increased P, total nitrogen, and total carbon contents in the soil.	Ibrahim et al., 2019
Wild mint (*Mentha arvensis*)	*Mentha arvensis* waste (450°C)	0, 2, and 4% (w/w)	Pot	Sandy loam soil	Cd, Pb	Biochar enhanced the Cd and Pb tolerance by decreasing Cd (50%) and Pb (25%) uptake in mint along with an increase in photosynthetic pigments and stomatal activity.	Nigam et al., 2019
White willow (*Salix alba*)	Carpinus betulus waste biomass (400°C)	0, 2.5, and 5% (w/w)	Pot	Sand	Cd, Cu, Pb	Biochar treatment increased the plant height, root length, leaf area, photosynthetic pigments, CO_2_ assimilation rate, and intracellular CO_2_ concentration in addition to reduced cd, Pb, and Cu availability.	Mokarram-Kashtiban et al., 2019
Lebbek tree (*Albizia lebbeck*)	Farmyard manure (450°C)	0, 3, and 6% (w/w)	Pot	Sandy loam	Cd	Application of biochar enhanced the growth and gas exchange characteristics by lowering the absorption rate of Cd in root, shoot, and leaves up to 34, 33, and 50% respectively.	Yousaf et al., 2019
Rice (*Oryza sativa*)	Rice straw (450°C)	0 and 1% (w/w)	Pot		Cd	Biochar treatment significantly decreased the Cd uptake in root (29%) and shoot (45%) along with a considerable increase in shoot and root dry weight of plant and chlorophyll-*a* concentration.	Rizwan et al., 2019b
Rice (*Oryza sativa*)	Sugarcane bagasse (500°C)	0 and 3% (w/w)	Pot	Fragile sandy soil	Cd	Application of biochar alleviates the ROS and decreased the bioavailability of Cd in fragile soil along with an increase in growth of plant root and photosynthetic pigments.	García et al., 2020
Wheat (*Triticum aestivum*)	Farm yard (500°C)	0, 2.5, and 5 g/kg of soil	Pot	Alkaline soil	Cd	Biochar application reduced the Cd concentration in plant root (71–92%), shoot (82–92%), and grain (90–96%) in addition to enhanced wheat yield.	Ijaz et al., 2020
Rapeseed (*Brassica napus*)	Woodchip (300°C)	0, 1, and 2% (w/w)	Pot		Cd, Pb, Ni, Cu	The concentration of Cd (44%), Pb (51%), Ni (59%), and Cu (45%) were decreased along with an increase in fresh root and shot biomass, total chlorophyll, and enzymatic antioxidant activity under biochar application.	Kamran et al., 2020
Quinoa (*Chenopodium quinoa*)	Wheat straw (350°C)	0, 1, and 2% (w/w)	Pot		Cd	The treatment with biochar enhanced the overall growth, pigments, and gas exchange parameters by limiting the Cd accumulation in root (30%), shoot (25%), and grain (45%) of quinoa.	Naeem et al., 2020
Wheat (*Triticum aestivum*)	Rice husk (400–500°C)	0.4, 3 and 5%	Pot		Cd, Pb	Biochar application showed a promising decrease in shoot Cd (77%) and Pb (50%) availability in the soil and increased the plant growth and grain yield.	Zhang S. et al., 2020
Cotton (*Gossypium hirsutum*)	Cotton straw (550°C)	0 and 3% (w/w)	Pot		Cd	Application of biochar considerably enhanced the chlorophyll contents, gas exchange parameters, and the activities of SOD and POD by decreasing the Cd uptake in both root (17.8%) and stem (15%).	Zhu et al., 2020
Radish (*Raphanus sativus*)	Wheat feedstock (500°C)	0 and 0.5% (w/w)	Pot	Paddy soil	Cd	Biochar application showed a prominent increase in the activity of antioxidant enzymes and mineral contents along with a clear reduction of 92% in Cd uptake through roots.	Dad et al., 2020
Rice (*Oryza sativa*)	*Platanus orientalis* branches (650°C)	0 and 3% (w/w)	Pot	Silty clay loam	Cd, As, Pb	Application of biochar reduced the bioavailability of Cd (37%) and Pb (23%) along with a considerable increase in catalase activity and grain yield.	Wen et al., 2020
Oak (*Quercus castaneifolia*)	Rice husk (500–550°C)	1, 3, and 5% (w/w)	Pot	Loamy soil	Cd	Biochar treatment improved the oak growth and decreased the bioavailability of Cd up to 67%.	Amirahmadi et al., 2020
Wheat (*Triticum aestivum*)	Dry maize (700°C)	0, 1.5, and 3% (w/w)	Pot		Cd	Plant fresh and dry biomass, root length, and root surface area were increased along with reduced Cd uptake in root (51%) and shoot (48%).	Jan et al., 2020
Tobacco (*Nicotiana tabacum*)	Tobacco stem (450°C)	0, 1, and 2% (w/w)	Pot		Cd	Application of biochar decreased the absorption, accumulation, and concentration of Cd in root (81%), stem (68%), and leaves (80%) along with increased plant biomass.	Yao et al., 2021
Sweet basil (*Ocimum ciliatum*)	Mulberry wood residues (530°C)	0, 1, and 2% (w/w)	Pot	Sandy loam	Cd	Biochar application reduced the Cd uptake in leaf up to 40% along with an increase in photosynthetic pigments, morphological traits, and catalase activity.	Mehdizadeh et al., 2021
Tobacco (*Nicotiana tabacum*)	Corn Cob (500°C)	0 and 1% (w/w)	Pot	Clay loam	Cd	Biochar treatment considerably reduced the Cd contents in shoot (32%) and improved the plant growth.	Erdem, 2021
Wheat (*Triticum aestivum*)	Bamboo biochar (750°C)	0, 0.1, 1, and 5% (w/w)	Pot		Cd	Cd uptake was reduced in root (34.06%), straw (21.57%), and grain (23.33%).	Ma et al., 2021
Tobacco (*Nicotiana tabacum*)	Peanut-shell waste (400°C)	0 and 1% (w/w)	Pot	Cinnamon soil	Cd	Photosynthetic pigments, gas exchange attributes, and activity of enzymatic antioxidants were increased along with a decrease of 14.8% in leaf Cd absorption.	Ren et al., 2021

Moreover, application of biochar enhances soil pH, water holding capacity, and porosity (Yuan et al., 2019). The role of biochar in growth enhancement is due to the presence of essential nutrients available for plants (Sohail et al., 2019). Rizwan et al. (2018) reported that application of biochar has a positive influence on plant growth. With application of biochar, the exchange portion of Cd in soils is reduced by up to 28% because of increase in soil pH (Bashir et al., 2018). Application of rice straw considerably reduced the concentration of extractable Cd and Cd in soils (Elyamine et al., 2019). Zia-ur-Rehman et al. (2020) reported that various types of biochar showed significant increase in wheat grain in addition to reduced Cd bioavailability. Different low-cost amendments enhanced the photosynthetic pigments and activity of enzymatic antioxidants (SOD, POD, CAT, and ASP) in maize (Shahkolaie et al., 2020).

#### Compost

In contrast to various fertilizers, utilization of compost is a valuable practice to increase soil fertility and crop yield (Abbas et al., 2020). Being a saturated organic carbon, compost enhances the HM-holding capacity of soils along with increase in soil absorption capacity (Medyńska-Juraszek et al., 2020). The bioavailability of HMs in soils has been decreased with the application of compost because of the mechanism of chelation, degradation of microbes, co-precipitation, and de-methylation (Hussain et al., 2021). Naturally, the process of composting helps to stabilize HMs and solid wastes from agricultural and municipal sources by degradation of various microbes (Awasthi et al., 2015). Addition of compost modifies soil aggregation, soil moisture content, and percentage of organic matter, and different nutrients resulted in higher crop growth and yield (Abd El-Mageed et al., 2018). This improved aggregation of soil resulted in altered soil physical and chemical properties, which helps to improve seed germination and roots of seedlings (Abd-El-Mageed et al., 2019). Addition of compost to soils showed 20, 19, and 10% decrease in shoot Cd, Cu, and Zn, respectively (Eissa, 2019). Application of vegetable waste compost reduced Cd concentration by up to 50 and 46% in maize shoots and roots, respectively, along with significant increase in plant growth and NPK contents (Bashir et al., 2021). An increase of 39–85 and 29–63% was observed in shoot and root fresh weight of pak choi cabbage, respectively, along with decrease in the concentration of Cd both in roots and shoots by up to 21–44 and 26–53%, respectively, when green waste compost was applied to HM-contaminated soils (Li et al., 2021).

#### Manure

Manure is used as a replacement for fertilizers, as it is a proper organic fertilizer for plant production (Zhen et al., 2020). Being an organic soil amendment, manure has been extensively used to mobilize HM-contaminated soils (Huang et al., 2018). Its application increased soil pH because of mineralization of carbon and excessive addition of basic cations (Hussain et al., 2021). Application of manure increased the organic matter in soils that resulted in reduced Cd mobilization and phytotoxicity (Liu et al., 2015). Higher accumulation of organic matter in soils stops Cd mobilization by forming organic compounds or, more importantly, adsorption (Halim et al., 2015). Long-term use of manure in uncontaminated soils introduced the availability of HMs as there is little or no information regarding the available or total HMs in the soil with the addition of manure in uncontaminated soils (Zhen et al., 2020). Li F. et al. (2019) stated that SOM had been increased in addition to reduced bioavailability of Cd with the addition of chicken manure in a Cd-contaminated paddy field. Farmyard manure application significantly decreased Cd bioavailability and increased wheat yield (Bashir et al., 2020). Treatment of contaminated soils with composted manure increased the growth of maize plants in addition to decreased HM uptake (Gul et al., 2016). Huang et al. (2020) observed a promising decrease in Cd and Zn bioavailability and enhanced biomass of *B. juncea* with the application of manure. Bashir et al. (2021) used animal manure to observe the growth of maize in polluted soil and found a prominent decrease of 58 and 52.4% in shoot and root Cd along with enhanced NPK contents.

### Genetic Strategies for Cadmium Remediation

**FIGURE 5:**
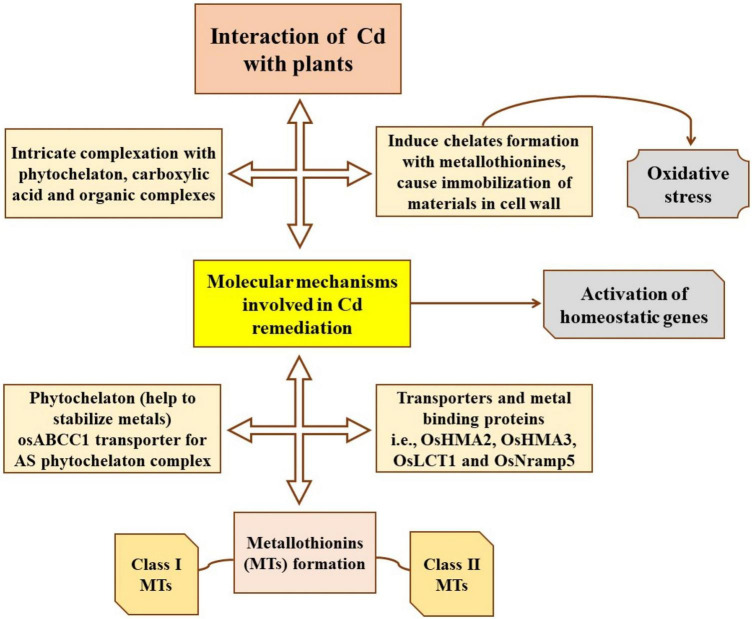
Schematic elucidation of molecular mechanisms involved in Cd remediation in plants.

Genetic engineering has played a vital role in improving the phytoremediation abilities of plants in terms of removing or detoxifying hazardous heavy metals in the environment. Several molecular techniques are being widely used to reduce Cd accumulation. Molecular mechanisms underlying Cd interactions and remediation are shown and described in Figure 5.

#### Improvement in Cadmium Tolerance by Harnessing Genetic Variation

Screening of genotypes for low Cd accumulation is the primary step to develop Cd-tolerant genotypes. Utilization of these genotypes will help in the development of transgenic crops with enhanced tolerance. Rice has a main role in the supply of Cd to humans, which causes Cd toxicity in them. The risk of Cd toxicity in human beings can be reduced by identifying rice genotypes with low Cd-accumulating capacity. Considerable genetic variability has been found in polished rice and brown rice ranging from 0.14-1.43 and 0.06–0.99 mg kg^–1^, respectively (He et al., 2006; Liu et al., 2007). Indica rice displayed higher Cd accumulation in shoots and grains than japonica rice (He et al., 2006; Uraguchi et al., 2009).

In cereals, a substantial amount of genetic variability has been observed in grain legume crops, i.e., soybean (Arao et al., 2003; Sugiyama et al., 2011; Salazar et al., 2012; Vollmann et al., 2015). High or low Cd accumulating genotypes were identified in different crops by analyzing genetic variations present among them. In wheat, genotypes with low Cd accumulation capacity included durum lines 8982-TL-L, CDC-Verona, and Strongfield (Clarke et al., 2002; Pozniak et al., 2009). On the other hand, high Cd accumulating wheat genotypes were Joppa and Carpio (Elias et al., 2015; Elias and Manthey, 2016). Introgression of genes from one plant to another is also a promising genetic engineering technique, as many plants express genes that help in Cd tolerance (Lan et al., 2012; Yuan et al., 2012; Menguer et al., 2013). Aegilops tauschii accessions, i.e., AS623194, AS623402, and AS623402, were recommended to be rich sources of Cd-tolerant genes, as wild species have high genetic variability for Cd accumulation. Identification and inclusion of these genotypes in crop improvement programs will be helpful in developing new Cd-tolerant cultivars. Genes or QTLs controlling the accumulation of Cd can be identified using these lines in mapping populations (Qin et al., 2015).

#### Genetic Engineering Techniques for Cadmium Tolerance

Genetic engineering along with the development of transgenic plants may help in the cultivation of crops in soils contaminated with Cd. Various approaches in genetic engineering can help in the identification of desirable genes that can be used to develop plants with increased HM tolerance. These genes may assist in translocation and deposition of metals in tissues of transgenic plants along with other characteristics that help in tolerance of metals (Mosa et al., 2016; Sheoran et al., 2016). Recent research has displayed that several genes are involved in regulation of HM tolerance. This may be performed either by overexpression of one gene or by overexpression of multiple genes simultaneously (Bhargava et al., 2012). Therefore, genetic modifications involving transfer or regulation of genes should be studied thoroughly to develop crops tolerant to HM stress (Gerszberg and Hnatuszko-Konka, 2017). Metallothioneins and phytochelatins are key components that can be utilized to protect plants against toxic metals. Several studies have shown the importance of these components in phytoremediation, as HM tolerance was increased in transgenic plants developed using metallothionein genes and synthases (Sunitha et al., 2012).

Gene manipulation is one the most important strategies for producing Cd-tolerant plants. It involves complete understanding of molecular mechanisms involved in Cd stress and can be utilized to develop methods to reduce damages that are caused by toxic metals. This may also help reducing Cd concentration in edible plant parts by breeding crop varieties with low Cd accumulation and more agronomical importance. Most effective molecular techniques used worldwide for Cd tolerance in crop plants have been discussed.

#### Quantitative Trait Loci Related to Cadmium Uptake and Accumulation

The identification of chromosomal regions governing genes that control Cd tolerance has become an important genetic tool with the discovery of quantitative trait loci (QTLs) using family based method. This method has helped in the illustration of underlying genetic basis of Cd accumulation in various crops (Ishikawa et al., 2005, 2010; Knox et al., 2009; Ueno et al., 2009a,b; Jegadeesan et al., 2010; Jha and Bohra, 2016). In rice, QTLs are supposed to be located on LGs 3, 6, and 8 in chromosome segment substitution lines (CSSLs) developed from the cross between Kasalath (indica) and Koshihikari (japonica) (Ishikawa et al., 2005).

A QTL responsible for Cd accumulation in roots and shoots was found on LGs 6 and 7, while LG 3 consisted of a QTL for root or shoot Cd content. LGs 1, 3, 5, and 8 governed a total of six QTLs responsible for Cd tolerance (Xue et al., 2009). In F2 resulting from a cross between Badari Dhan and Shwe War, it was demonstrated that QTLs controlling 16.1% of total phenotypic variation (PV) related to Cd tolerance was mapped on LG 11 (Ueno et al., 2009a). QTL *qLCdG11* for Cd tolerance was also mapped on LG 11 in an RIL population developed from a cross between Fukuhibiki 9 and LAC23. Major QTLs elucidating 85% of PV related to Cd accumulation were reported on LG 7 in rice (Ueno et al., 2009b). Afterward, QTL *qGCd7* found on LG 7 explained 35.5% of phenotypic variation by analyzing backcross inbred lines developed by crossing Sasanishiki (japonica) with Habataki (indica). Major QTL *qlGCd3* controlling Cd levels in rice grains was discovered by mapping CSSLs developed from a cross between LAC23 and Koshihikari (Abe et al., 2013). A total of 18 Cd-accumulating QTLs in milled rice and 14 in brown rice were reported to be located on LGs 2, 3, 4, 5, and 7 (Da-wei et al., 2018).

Luo et al. (2018) demonstrated CAL1 as a QTL in rice causing 13% change in Cd accumulation in leaves of a double haploid population. CAL1 is responsible for Cd translocation from root to shoot, and rice mutants with CAL1 knockout displayed decreased Cd concentration in leaves (Luo et al., 2018). Cd-accumulating QTLs were mapped in rice by growing an inbred population (743/Katy) derived from Xiang in soil contaminated with Cd revealing two QTLs, i.e., qCd-7 and qCd-2 involved in the process of Cd uptake and accumulation in rice (Li W. et al., 2016; McCouch et al., 2016). Sato et al. (2011) discovered two QTLs in brown rice affecting Cd accumulation, i.e., qLCdG11 and qLCdG3, explaining 9.4–12.9 and 8.3–13.9% of phenotypic variation, respectively. Five most important QTLs were discovered; three of them (gcc3, gcc9, and gcc1) were responsible for grain Cd concentration; scc10 was linked with Cd accumulation in shoots, and the QTL sgr5 was involved in supply of Cd to roots and shoots. Grain Cd accumulation was highly influenced by Srg5 in rice (Abe et al., 2013).

In durum wheat, the gene *Cdu1* plays a role in Cd uptake, as reported by Knox et al. (2009), whereas Wiebe et al. (2010) illustrated the role of *Cdu1* in accumulation of Cd in wheat grains. Recently, a major QTL on 5BL has been located in durum wheat controlling 54.3% PV for Cd uptake with the help of high-density genotyping (AbuHammad et al., 2016). In an RIL population developed by crossing Divide with D041735, another QTL, “*QCdu. ndsu-5B,”* has been discovered on 5B for Cd intake and tolerance. In soybean, a QTL for lower accumulation of Cd has been mapped on LGK controlling up to 57.3% of phenotypic variation (Jegadeesan et al., 2010).

#### Association Studies to Check Plant Response to Cadmium Stress

Advancement in methods used for genome mapping has led to revelation of various significant market trait associations (SMTAs) present in genomes for several traits including Cd intoxication (Huang and Han, 2014). Additionally, elucidation of crop genomes and other techniques, such as genome resequencing and genotyping by sequencing (GNB) to identify SNPs has helped in performing GWAS to study complex plant traits, i.e., HM toxicity and tolerance. Six SMTAs (such as pdil5-1, TaAP2-B, TaAP2-D, DME-5A, and Acc-1) related to genes controlling Cd tolerance were revealed in 235 accessions of *Aegilops tauschii* by GWAS using 7,185 SNPs.

A total of 17 QTLs for grain Cd accumulation were discovered in rice by GWAS conducted on 276 rice accessions using 416 K SNPs (Liu et al., 2019). A total of 312 out of 1,568 accessions of rice displaying more diversity were selected and evaluated to develop low Cd accumulating germplasm (Li W. et al., 2016; McCouch et al., 2016). Twenty-four rice accessions including 3 Indica accessions linked with reduced accumulation of Cd by up to 0.2 mg kg–1 were selected based on GWAS. A total of 312 accessions including subpopulations of Indica and Japonica rice were analyzed, leading to discovery of 28 QTLs related to Cd uptake and accumulation in plant tissues. Genes, i.e., OsNRAMP5, OsNRAMP1, OsLCD, and OsHMA3, that have been already discovered were also reported in recent GWASs (Li W. et al., 2016; McCouch et al., 2016).

A GWAS was carried out on 100 barley accessions and resulted in identification of several QTLs, i.e., 9 for root Cd accumulation, 21 for shoot Cd, 14 for transport of Cd from roots to shoots, and 15 for grain Cd amassment (Wu et al., 2015). Recently, 63 SMTAs on 5 LGs for leaf Cd were discovered in maize by GWAS using 43,737 SNPs (Zhao et al., 2018). This study also revealed several genes such as GRMZM2G45549 and GRMZM2G124103 coding for vacuolar ATPase, and GRMZM2G175576 for ATPase controlling Zn/Cd transport. Identification of specific DNA markers and accurate phenotyping techniques for study of Cd stress in crop plants will help in better understanding of Cd accumulation mechanism and improvement of genotypes for Cd tolerance.

#### Cadmium Phytoremediation by Overexpression of Genes

Introduction and overexpression of genes associated with metal uptake and translocation are the most effective and commonly used methods for phytoremediation using transgenics (Shukla et al., 2013; Mani and Kumar, 2014; Das et al., 2016). Various pathways including metal intake by roots, formation of metal-ligands and metal-chelator complexes, deposition of metals in vacuole, and long-distance translocation to shoots through symplast and apoplast can be exploited to enhance metal accumulation in plant tissues, as metal intake is a complex process (Nakamura et al., 2014; Das and Jayalekshmy, 2015). Genes coding for metal ion transporters and chelators are being used to manipulate plant metal uptake and translocation.

Metal transporters are also linked to gene families coding for ZIP proteins that are involved in Fe and Zn transport in cytoplasm. Conolly et al. (2002) reported 150% enhanced accumulation of Cd and Zn when gene coding for AtIRT1 was overexpressed in *A. thaliana*. However, some ZIP genes are highly specific, and their overexpression does not lead to improved accumulation of HMs like Cd (Tiong et al., 2014). Therefore, another strategy has been exploited in which genes coding for metal transporters and involved in microelements uptake and transport are transformed into plants. When absorption microelements are increased, HM uptake is reduced. These genes manipulate plant’s ability to collect HMs by enhancing or decreasing the effect of ZIP genes. This method may be utilized for phytoremediation of soil contaminated with HMs and for biofortification of crops (Siemianowski et al., 2014; Gong et al., 2015).

Nakamura et al. (2014) developed transgenic plants of *Nicotine tabacum* by overexpression of cysteine synthase (CS) and serine acetyltransferase (SAT) involved in cysteine biosynthesis. The plants produced by overexpression of these genes displayed enhanced Cd tolerance. He et al. (2016) inserted a homologous corn gene in *Arabidopsis* by activating methyltransferase gene (CIMT1 protein). Results displayed enhanced Cd tolerance in *Arabidopsis* by the overexpression of this gene. This gene could be utilized to enhance Cd tolerance in agricultural crops. Lee and Back (2017) overexpressed melatonin-related genes using transgenic OsSNAT1 in rice. The study revealed that transgenic OsSNAT1 provides tolerance to Cd. An expansin gene, TaEXPA2, was isolated from wheat and overexpressed in tobacco plants. This enhanced tolerance to Cd toxicity in tobacco plants and improved seed germination and growth of seedling and roots (Ren et al., 2018).

Expression of OXS3 (OXIDATIVE STRESS 3)-like gene fragments led to reduced accumulation of Cd in rice grains and other plant tissues without affecting yield and amount of minerals, i.e., Cu, Mn, Zn, and Fe (Blanvillain et al., 2009). Hence, this approach seems to be promising for production of Cd-tolerant genotypes without any loss in yield or minerals. OsHMA3 was involved in the transport of Zn to the vacuoles in root cells (Sasaki et al., 2012). The expression of OsHMA3 along with the OsHMA2 promoter had no effect on the growth of plants in vegetative stage. Moreover, when OsHMA3 was expressed under OsHMA2 control, the expression and localization of OsHMA3 in tissues were increased, and significant reduction in Cd concentration was observed because of increased Cd sequestration in roots, nodes, and vacuoles (Shao et al., 2018).

The functional gene OsHMA3 from rice was overexpressed in wheat for Cd sequestration in roots. Cd accumulation in roots was enhanced by up to 110–125%. Reduction in Cd accumulation in wheat grains was observed to be up to 40-folds, while translocation of Cd from roots to shoots was decreased by approximately 10-folds (Zhang L. et al., 2020). This technique offers an effective solution to reduce Cd accumulation in plants, leading to reduced health risks.

##### Clustered Regularly Interspaced Short Palindromic Repeats/Cas9-Based Genome Editing to Reduce Cadmium Accumulation

Genome editing tools like clustered regularly interspaced short palindromic repeats (CRISPR) and CRISPR-associated protein 9 (Cas9) systems (CRISPR/Cas9) can be used for Cd tolerance in plants, i.e., rice (Ishizaki, 2016; Jung et al., 2018). It is possible to develop genotypes with low Cd accumulation without affecting plant growth and yield by developing mutants with transporters having low Cd affinity, such as OsNramp5 and OsLCT1, using the CRISPR/Cas9 technology. Recently, another approach has been discovered that may be helpful in getting desired results, and it involve deletion of large DNA fragments using CRISPR/Cas9.

## Conclusion

Higher uptake of Cd leads to toxicity in plants, and reduction of Cd uptake by plants is also imperative for ensuring food safety. As a result, plants that are confined in their habitat are more likely to be exposed to Cd toxicity, which negatively impacts all growth- and yield-related characteristics, resulting in substantial economic losses. In this review, we discussed that plants have an efficient biochemical defense mechanism and utilize numerous physiological and molecular processes to deal with increased Cd exposure to offset its inhibitory effects. Exclusion of excess Cd at the root level, vacuolar sequestration, enzymatic detoxification, and preservation of essential cations are some of the techniques utilized to reduce Cd^2+^, which are significant disadvantages of Cd toxicity. Cd toxicity can also be reduced using various remediation techniques such as phytoremediation, PGPR inoculation, microbial-assisted remediation, chemical remediation, nutrients utilization, and organic amendments. Moreover, through genetic engineering and molecular breeding, capacity for remediation and tolerance to Cd toxicity can be improved. This review covers all probable processes of Cd toxicity remediation approaches; however there are still some information gaps. As a result, further research is needed to investigate Cd toxicity at the grain level, as well as its impacts on other living biota and the mechanisms of its tolerance at the cell and organelle levels. Significant and consistent QTLs for Cd tolerance were discovered in mapping populations with various genetic origins, as well as chosen donors, which indicated a significant promise for application in improved breeding methods. Although many efforts have been made to reduce Cd toxicity in plants, further research should be carried out by keeping these points in focus. There is a need to identify Cd toxicity pathways at molecular levels for better understanding of Cd toxicity. More detailed studies are required to understand the mechanism of different amendments in reducing Cd toxicity in plants. There is a need for a multidisciplinary approach to identify key target tolerance traits with improved high-throughput screening techniques that can provide valuable insights into the underlying mechanisms of Cd tolerance and homeostasis, especially in agronomic crops, for a better understanding of the genetics of Cd tolerance. To draw a sound conclusion, long-term field trials are required for estimation of a risk and benefit analysis for various management strategies.

## Author Contributions

UZ, WJ, and AM: conceptualization. WX and MN: methodology. JK and SH: software. MA: resources. MM and MK: data curation. UZ and FH: writing (original draft preparation). AM, JK, MB, MN, WX, and MI: writing (review and editing). NA and NF: visualization. MI and SH: supervision. MB and MK: project administration. All authors read and agreed to the published version of the manuscript.

## Conflict of Interest

The authors declare that the research was conducted in the absence of any commercial or financial relationships that could be construed as a potential conflict of interest.

## Publisher’s Note

All claims expressed in this article are solely those of the authors and do not necessarily represent those of their affiliated organizations, or those of the publisher, the editors and the reviewers. Any product that may be evaluated in this article, or claim that may be made by its manufacturer, is not guaranteed or endorsed by the publisher.
